# Thiol post‐translational modifications modulate allosteric regulation of the OpcA–G6PDH complex through conformational gate control

**DOI:** 10.1002/pro.70561

**Published:** 2026-04-13

**Authors:** Hoshin Kim, Song Feng, Pavlo Bohutskyi, Xiaolu Li, Daniel Mejia‐Rodriguez, Tong Zhang, Wei‐Jun Qian, Margaret S. Cheung

**Affiliations:** ^1^ Physical Sciences Division, Physical and Computational Sciences Directorate, Pacific Northwest National Laboratory Richland Washington USA; ^2^ Biological Sciences Division, Earth and Biological Sciences Directorate, Pacific Northwest National Laboratory Richland Washington USA; ^3^ Department of Biological Systems Engineering Washington State University Pullman Washington USA; ^4^ Environmental Molecular Sciences Laboratory Richland Washington USA; ^5^ University of Washington Seattle Washington USA

**Keywords:** allosteric regulation, oxidative pentose phosphate pathway, PTM‐psi, redox post‐translational modification, redox proteomics, regulatory protein–protein interactions

## Abstract

In cyanobacteria, the redox‐sensitive protein OpcA acts as a metabolic switch for G6PDH, enabling rapid adjustment of reducing power generation from glycogen catabolism and thereby precisely regulating carbon flux between anabolic and catabolic pathways. Although redox‐sensitive cysteines in OpcA are known to regulate G6PDH, the mechanisms by which redox post‐translational modifications (PTMs) on OpcA control G6PDH structure and activity remain unclear. Here, we combine computational modeling with experimental redox proteomics in *Synechococcus elongatus* PCC 7942 to dissect this mechanism. Experimentally, redox proteome analysis revealed differential redox PTM patterns, particularly on cysteines within the G6PDH‐binding site of OpcA. These environmentally sensitive PTM changes at the interface suggest that thiol modifications in this region form a key regulatory node. More broadly, redox proteomics identified site‐specific cysteine modifications under light/dark transitions and circadian cycling, linking distinct redox regimes to discrete PTM states. We employed PTM‐Psi simulations to show that thiol PTMs near the OpcA–G6PDH interface are critical for allosteric regulation of G6PDH. The thiol PTMs on OpcA affect a putative gate region in G6PDH for substrate ingress and product egress as well as key hydrogen‐bond networks within the active site. We infer that PTMs on OpcA tune the conformational landscapes of individual G6PDH subunits toward functionally relevant configurations according to environmental gradients, biasing the enzyme toward catalytically favorable states. Together, our results reveal a molecular mechanism in which thiol PTMs on OpcA modulate G6PDH structure and function through PTM‐induced reorganization of conformational dynamics and allosteric communication. These findings demonstrate that PTM‐level regulation provides a critical control layer from genotypes to phenotypes that enables cyanobacteria to rapidly adapt to environmental fluctuations through precise metabolic fine‐tuning.

## 1 INTRODUCTION

In cyanobacteria, the oxidative pentose phosphate pathway (OPPP) serves as a critical metabolic route for generating NADPH and biosynthetic precursors (Diamond et al., 2017; Yang et al., 2002), particularly during periods when photosynthetic reducing power is unavailable (e.g., dark conditions) or when cells initiate the restart of photosynthesis (Makowka et al., 2020; Shinde et al., 2020). This pathway is essential for maintaining cellular redox homeostasis when photosynthesis cannot provide sufficient NADPH and biosynthetic intermediates for cellular processes and antioxidant systems. The first and rate‐limiting enzyme of this pathway, glucose‐6‐phosphate dehydrogenase (G6PDH), is subject to intricate regulation to ensure adequate NADPH production while coordinating carbon flux between the OPPP and the Calvin‐Benson‐Bassham (CBB) cycle, thereby maintaining optimal reducing power balance and biosynthetic intermediate availability under varying environmental conditions (Doello et al., 2025; Ito & Osanai, 2020). A key regulatory mechanism involves the redox‐sensitive oxidative pentose phosphate cycle protein (OpcA), which modulates G6PDH activity in response to the cellular redox state (Doello et al., 2024; Née et al., 2013). During the daytime, when photosynthesis provides abundant reducing power and the CBB cycle is active, OpcA forms a reduced state due to the action of the thioredoxin (Trx) system that obtains electrons from the ferredoxin pool. In this reduced form, OpcA inhibits G6PDH, minimizing the activity of the OPPP to prevent unnecessary NADPH production and allowing only the minimal flux needed to sustain the OPPP shunt, which provides essential metabolic intermediates for biosynthetic processes while directing photosynthetically produced sugars into glycogen storage under stable light conditions. At night, however, cells rely on glycogen breakdown for energy, and the absence of photosynthetic electron transport leaves the Trx system in an oxidized state without reduced ferredoxin. Under these conditions, OpcA shifts to its oxidized form, which is critical for the rapid activation of cellular reducing power generation through the OPPP (Doello et al., 2025).

Recent structural studies have shed light on the molecular basis of this regulation. Cryo‐electron microscopy (cryo‐EM) analyses revealed that oxidized OpcA binds to the G6PDH tetramer, inducing conformational changes that dramatically enhance enzymatic activity to meet the cell's immediate demand for reducing power (Doello et al., 2024). Conversely, when photosynthetic reducing power becomes available, reduction of OpcA by the light‐activated Trx system disrupts this interaction, leading to decreased G6PDH activity and effectively shutting down NADPH production via the OPPP. This redox‐dependent modulation creates a sophisticated reducing power management system: under light conditions, photosynthetic electron transport provides abundant NADPH, allowing the CBB cycle to operate with minimal flux through the OPPP shunt while directing the majority of fixed carbon toward glycogen storage for future energy and reducing power needs (Johnson et al., 2025).

The redox sensitivity of OpcA is primarily mediated by intramolecular disulfide bridges involving conserved cysteine residues. The recent cryo‐EM study of the OpcA‐G6PDH complex from the cyanobacterial strain *Synechocystis* sp. PCC 6803 revealed that intramolecular disulfide bond formations in OpcA induce structural changes in the active site of G6PDH, enhancing its affinity for glucose‐6‐phosphate and increasing its enzymatic activity (Doello et al., 2024). Mutagenesis and mass spectrometry analyses have identified two cysteine residues, C393 and C399, in OpcA from *Anabaena* sp. PCC 7120 as critical residues for this redox regulation (Mihara et al., 2018). Reduction of these disulfide bonds by thioredoxin leads to conformational alterations that prevent OpcA from effectively binding G6PDH, thereby rapidly attenuating its activity and shutting down NADPH production when reducing power is abundant from photosynthesis. These findings establish the molecular foundation for understanding how specific cysteine residues function as redox‐sensitive switches in metabolic regulation. Interestingly, in heterocysts—specialized nitrogen‐fixing cells in filamentous cyanobacteria—thioredoxin target proteins, including OpcA, remain more oxidized even under light conditions. This adaptation allows G6PDH to remain active in heterocysts during daylight, ensuring a continuous supply of reducing power for the highly energy‐demanding process of nitrogen fixation (Mihara et al., 2019). This specialized regulation demonstrates how cyanobacteria have fine‐tuned the OpcA‐G6PDH system to meet the distinct metabolic demands of different cell types within the same organism.

Despite these insights, experimentally capturing the transient and reversible nature of redox PTMs remains challenging. Traditional structural biology techniques, such as X‐ray crystallography and nuclear magnetic resonance (NMR) spectroscopy, often fail to resolve the dynamic conformational states induced by PTMs, especially when modifications occur at low stoichiometry or are transient (Chapman et al., 2023; Keedy et al., 2015). Mass spectrometry excels at identifying PTM sites but lacks the capacity to provide detailed structural information (Azevedo et al., 2022). These limitations necessitate complementary approaches to fully understand the structural and functional ramifications of redox PTMs, particularly for understanding how these modifications enable rapid and targeted metabolic responses to environmental changes that are essential for cyanobacterial survival in fluctuating conditions.

Mass spectrometry‐based techniques have advanced to enable site‐specific and stoichiometric analyses of cysteine thiol modifications, such as *S*‐glutathionylation, *S*‐nitrosylation, and disulfide bond formation (Li et al., 2021; Li et al., 2022). Innovative methodologies facilitate the enrichment and detection of reversibly oxidized cysteine residues, thereby illuminating the redox landscape within cells and tissues (Guo, Gaffrey, et al., 2014; Li et al., 2023). These proteomic strategies are particularly valuable for uncovering redox‐sensitive proteins and pathways that may be elusive to traditional biochemical assays (Duan et al., 2017; Fu et al., 2020; Sarkar et al., 2025; Zhang et al., 2021) and have proven especially powerful for characterizing dynamic redox changes in cyanobacteria during light/dark transitions that drive metabolic reprogramming. The computational workflow of Post‐Translational Modification on Protein Structures and their Impacts on dynamics and functions (PTM‐Psi) has been developed to leverage molecular dynamics (MD) simulations and advancements in force field development as a powerful tool to bridge this knowledge gap regarding how chemical modifications, such as PTMs, affect protein dynamics (Mejia‐Rodriguez et al., 2023). The PTM‐Psi toolkit streamlines the parameterization of oxidized cysteine residues with quantum chemistry packages, enabling the investigation of their impact on protein structure and function. By providing atomistic insights into protein dynamics, MD simulations allow for the exploration of conformational landscapes inaccessible to static experimental methods. Furthermore, methods combining fluctuation relations with MD simulations have been developed to calculate redox potential changes in proteins, offering a computational approach to study redox regulation mechanisms (Mejia‐Rodriguez et al., 2023; Oliveira et al., 2024). Complementing MD simulations, redox proteomics has become an indispensable approach for the comprehensive identification and quantification of redox PTMs across the proteome.

Here, we implement the complementary approach of redox proteomics and PTM‐Psi to elucidate the conformational transitions associated with the redox state of OpcA and its binding affinity to G6PDH in the binary OpcA–G6PDH complex. Such studies provide valuable insights into the allosteric mechanisms governing the OPPP in cyanobacteria by revealing how rapid environmental responses are achieved through precise molecular switches operating on sub‐second timescales to maintain cellular reducing power homeostasis. Moreover, integrating MD simulations with experimental data can facilitate the development of predictive models for redox regulation, with potential applications in metabolic engineering and synthetic biology. The dynamic interplay between redox PTMs and protein function, exemplified by the OpcA–G6PDH complex, highlights how computational approaches like MD simulations contribute to unraveling intricate regulatory mechanisms evolved in phototrophs for rapid response to environmental fluctuations to maintain metabolic efficiency. This understanding opens new avenues for developing predictive redox regulation models to enable rational metabolic interventions, with broad therapeutic and industrial applications.

## 2 RESULTS

### 2.1 Thiol PTMs within OpcA modulate protein structure and flexibility to enable metabolic switching

**FIGURE 1:**
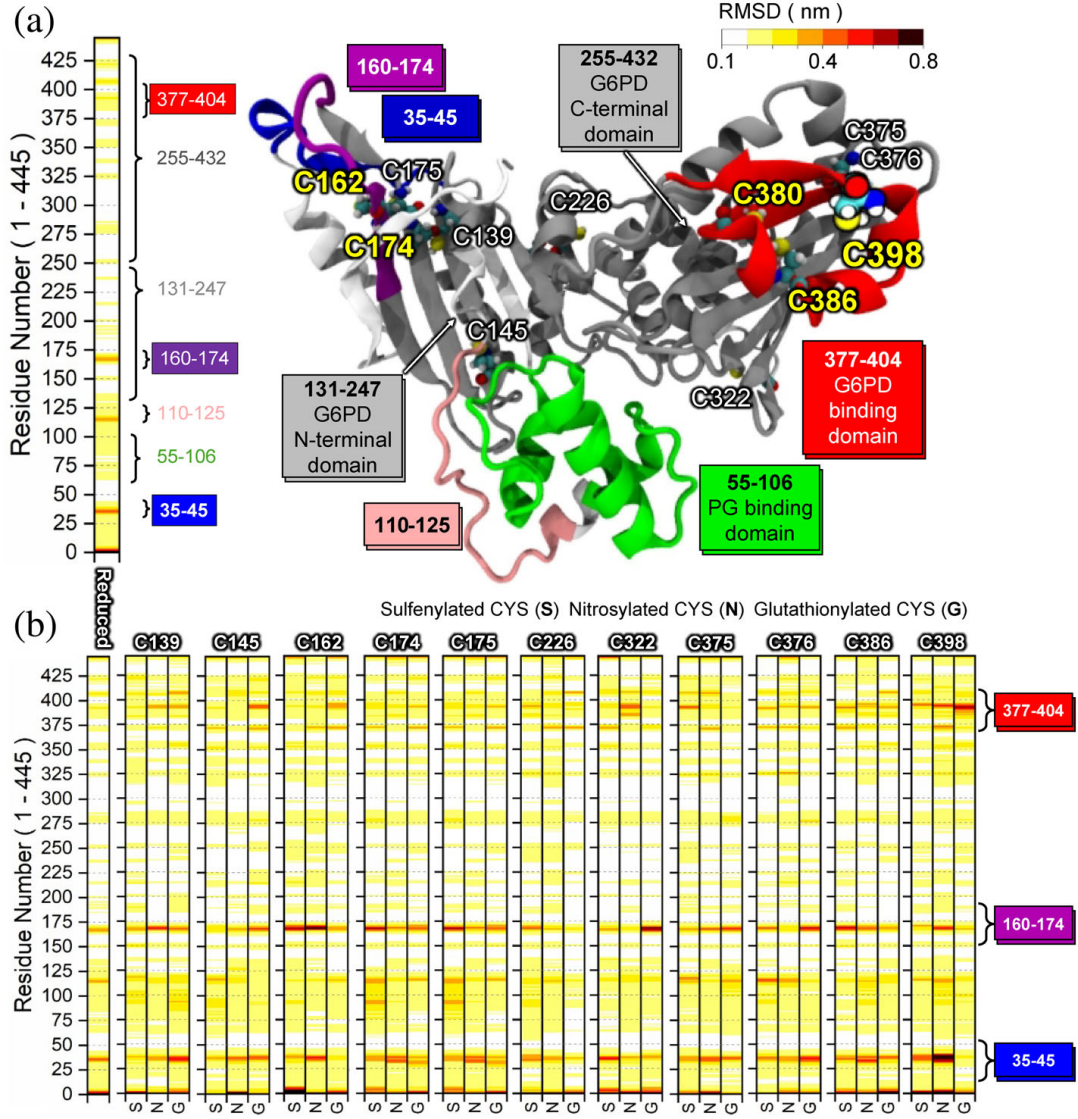
(a) RMSF analysis of the reduced OpcA showing regional flexibility patterns. A representative snapshot highlights putative functionally important regions: (I) peptidoglycan (PG) binding site (green), (II and III) N and C terminal domains (gray), (IV) G6PDH binding site (red). Flexible regions identified by MD simulations are marked in blue, pink, and purple. (b) RMSF comparison between reduced and PTMed OpcA, where specific cysteine sites were replaced with sulfenylated (S), nitrosylated (N), or glutathionylated (G) cysteines. Darker red indicates higher RMSF and more flexible regions.

The OpcA protein in *S. elongatus* comprises 445 amino acids (Figure S1) and contains four functionally important regions: (I) a peptidoglycan (PG) binding region (residues 55–106), (II) the G6PDH‐binding N‐terminal domain (residues 131–247), (III) the G6PDH‐binding C‐terminal domain (residues 255–432), and within this C‐terminal domain, (IV) the binding sites (residues 377–404) for G6PDH (Figure 1a). Regions (I–III) were annotated in UniProt (ID: Q54709) (Consortium TU, 2024) and (IV) was identified by a recent cryo‐EM study (Doello et al., 2024). Notably, all cysteine residues in the OpcA protein are strategically positioned within the putative G6PDH binding domain (Figure 1a), suggesting that redox‐dependent oxidation of these cysteine residues directly modulates the formation and stability of the binary G6PDH‐OpcA complex to control cellular reducing power generation through the OPPP.

We first performed all‐atom MD simulations of the OpcA protein as a monomer with various cysteine PTMs to elucidate how these modifications might affect its structure and dynamics in the context of G6PDH regulation. In the case of OpcA with reduced cysteines (reduced case), three flexible regions were observed, corresponding to amino acids ranging from 35–45, 110–125, and 160–174 (blue, pink, and purple, Figure 1a). These loops are named loop 35–45, loop 110–125, and loop 160–174. Interestingly, our MD simulation results demonstrated that the region corresponding to the potential G6PDH binding site becomes structurally affected when three different types of PTM are applied to the cysteine sites, which were not observed in the reduced case (highlighted in red in Figure 1b). For example, this G6PDH binding site exhibited relatively large fluctuations when C398 was replaced with its glutathionylated form.

**FIGURE 2:**
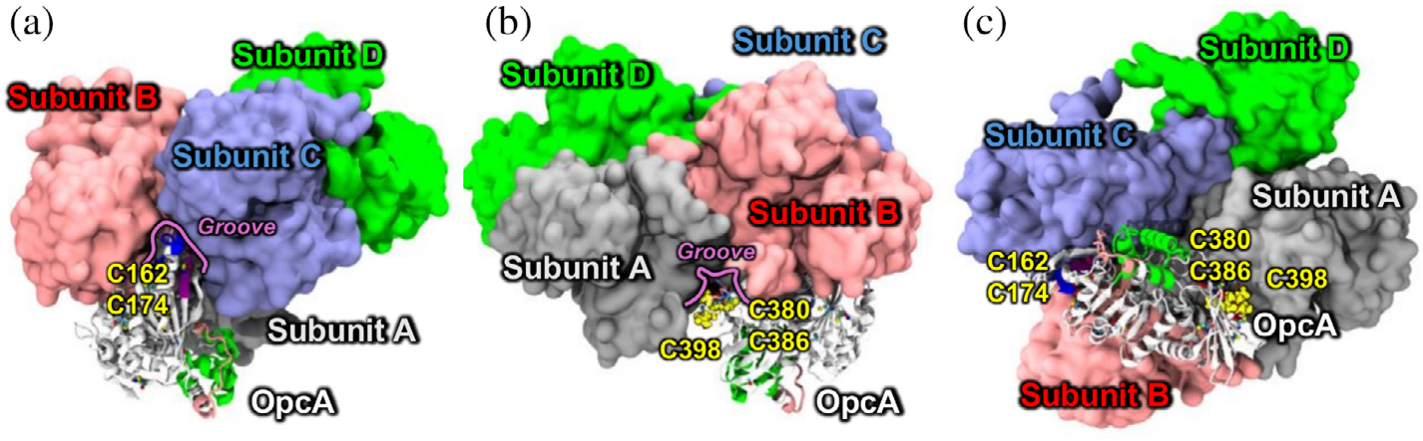
A predicted structure of the OpcA–G6PDH complex with four subunits A, B, C, and D shown as a side view. (A and B) Side views highlighting close contacts between flexible loops 35–45 (blue) and 160–174 (purple), the putative G6PDH binding site, region IV (residues 377–404, red) in OpcA, and the G6PDH tetramer. Grooves between two G6PDH subunits that establish close contact with OpcA are labeled with “Groove”. (C) Bottom view showing the OpcA monomer in contact with three G6PDH subunits (A, B, and C), but not with the fourth subunit, D.

We predicted the structure of the OpcA‐G6PDH binary complex using AlphaFold3 (Abramson et al., 2024) to understand how OpcA interacts with the G6PDH complex, and to explore how possible thiol PTMs on cysteines can impact these protein–protein interactions (Figure 2). In this model, the binary complex is composed of four G6PDH subunits and one OpcA monomer, because OpcA monomers are known to interact with either a dimer of two identical G6PDH monomers or a tetramer of two dimers, depending on the conditions (Au et al., 2000; Kiani et al., 2007). Moreover, the only available 3D structure of this complex that can be compared with our model is from different cyanobacteria and consists of the tetrameric form (comprised of identical subunits labeled with A, B, C, D) of G6PDH complexed with an OpcA monomer (Doello et al., 2024). The predicted monomeric structures of OpcA and G6PDH, as well as their complex form, showed high structural similarities when compared with the experimentally verified 3‐D structures of OpcA (Doello et al., 2024) and G6PDH (Doello et al., 2024; Wei et al., 2022) (Figure S2). The predicted structures highlighted that the flexible regions influenced by PTMs, including loops 35–45 and 160–174 (highlighted in blue and purple in Figure 1, respectively) and the putative binding region (Region IV, boxed in red in Figure 1), are located near the interfacial region between G6PDH and OpcA. Specifically, the two flexible regions are positioned in the vicinity of the grooves of subunits A and C (Figure 2a), while the putative binding region forms interactions at the grooves of subunits A and B (Figure 2b). Additionally, the OpcA monomer makes close contact with three G6PDH subunits (A, B, and C), while no direct contact with subunit D was observed (Figure 2c). Given that the amino acids within these flexible regions and putative binding sites are indeed involved in the interactions with the G6PDH complex, we hypothesize that PTMs of the cysteine residues located within the two spatially separated regions (loop 160–174 and Region IV 377–404, the binding sites with G6PDH) could affect the structure and function of the whole G6PDH complex.

### 2.2 Redox proteomics data and computational analyses reveal the specific cysteine locations and types of PTMs that occur under physiological conditions

**FIGURE 3:**
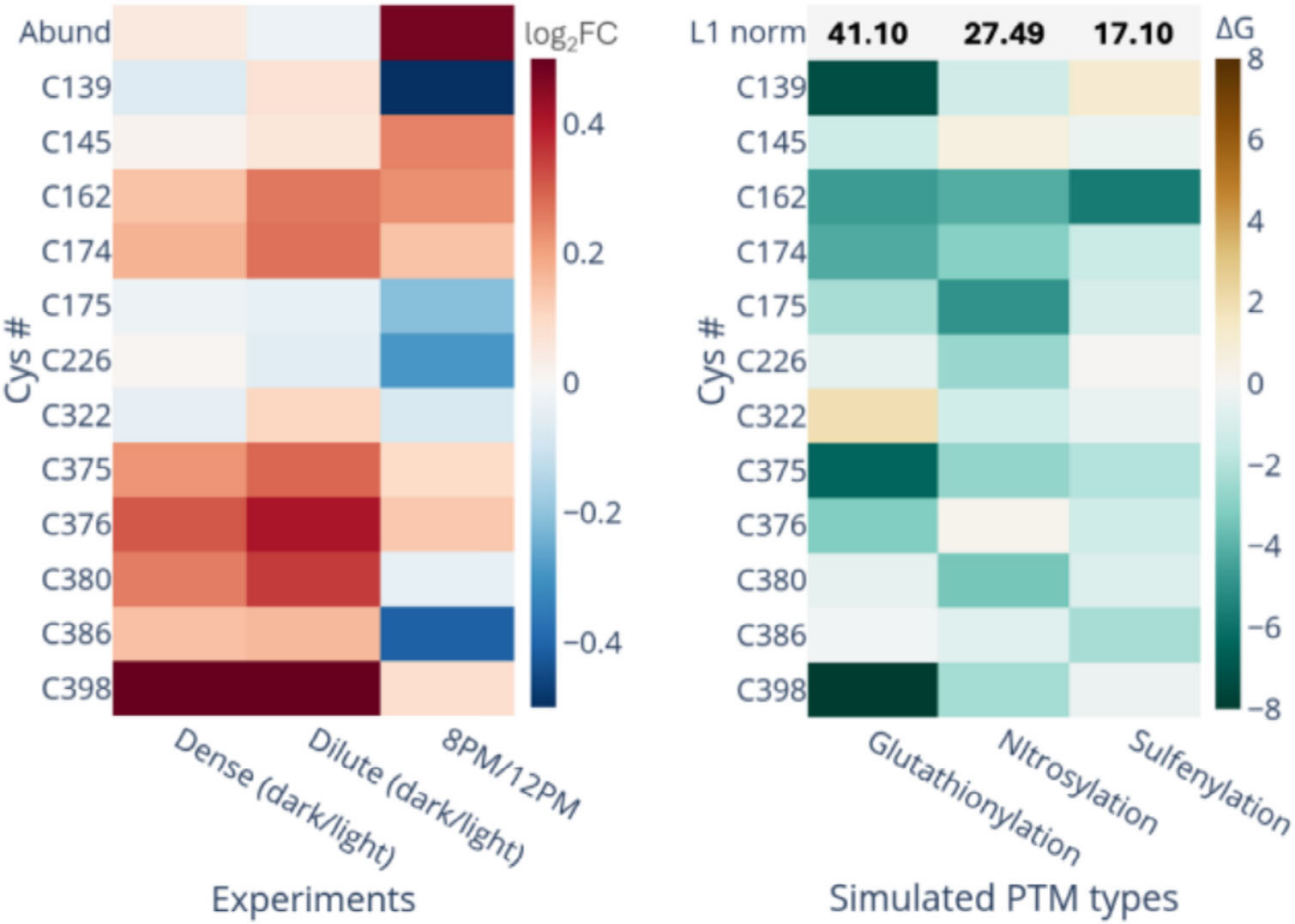
Experimental redox proteomics and computational free energy analysis of cysteine thiol PTMs in OpcA. (Left Panel) Changes in oxidation levels of individual cysteine sites and OpcA protein abundance (labeled as “Abund”) measured by redox proteomics. Heatmap shows Log2 fold changes (log_2_FC) in cysteine oxidation when light‐adapted cyanobacterial cells are switched to dark for 2 h either under dense (high cell density with increased self‐shading) or dilute (low cell density with reduced self‐shading) culture conditions, and when comparing diel cycle‐adapted cells at dusk (8:00 p.m.) versus noon (12:00 p.m.) under similar light conditions. Blue indicates a decrease; red indicates an increase in oxidation. (Right Panel) The relative binding free energy (RBFE) for transformation from three different PTM‐modified cysteine to reduced cysteine. More negative values indicate that a given PTM is energetically more favorable. The L1 norm is the calculated Manhattan distance of all the free energy changes from site modifications of corresponding PTM types. This L1 norm measures the overall effects of different PTM types on all captured sites.

To explore how light/dark perturbations and circadian rhythms affect the PTMs of these cysteines, we investigated all cysteine sites in OpcA using experimental redox proteomics data. The light‐to‐dark transition experiments were performed using both dense and dilute cultures adapted to continuous light conditions, ensuring that the observed redox changes are directly attributable to light availability without impact from circadian regulation. As illustrated in Figure 3, our redox proteomics data demonstrated that cysteines in flexible regions, including C162 and C174 (loop 160–174, see Figure 1a) and C380, C386, and C398 (the putative G6PDH binding region, Region IV, see Figure 1a), are sensitive to light‐to‐dark transitions and become increasingly oxidized under dark conditions when photosynthesis‐driven reducing power becomes unavailable (Figure 3a). Importantly, OpcA protein abundance remained unchanged during the 2‐h light‐to‐dark transition in both dense and dilute cultures, indicating that PTM‐based regulation operates independently of protein expression changes during short‐term light perturbation. Cysteine residues C162–C174 and C380–C386 can form disulfide bonds (Figure S1) and, according to a recent cryo‐EM study, these disulfide bonds allosterically influence the structure of the G6PDH complex and thus play a pivotal role in its functionality (Doello et al., 2024). Notably, experimental data indicated that changes in oxidation of these cysteine residues were more pronounced when cells were grown in dilute cultures, where individual cells experienced higher light irradiance per cell due to reduced self‐shading before transitioning to dark conditions. Furthermore, we discovered that glutathionylation at C398 (Region IV) is the most energetically favorable PTM compared to other potential thiol PTM sites during light/dark perturbations. This cysteine residue is not conserved in OpcA proteins from other species, highlighting its specificity to *S. elongatus* (Figure S3).

To examine the influence of circadian regulation beyond immediate light/dark responses, we compared the redox state of OpcA cysteines in diel cycle‐adapted cells at two different circadian time points under identical light conditions. Importantly, in contrast to the short‐term light/dark experiments where OpcA protein abundance remained unchanged, OpcA protein abundance showed significant changes between dusk and noon samples, revealing that abundance‐based regulation operates on circadian timescales to modulate OpcA function. The redox state of cysteine residues showed distinct patterns when comparing dusk (8:00 p.m.) and noon (12:00 p.m.) samples, demonstrating clear differences between the light/dark transition experiments. C162 and C174 (loop 160–174) showed higher oxidation at dusk (8:00 p.m.) compared to noon (12:00 p.m.), while C380, C386, and C398 (Region IV) exhibited more complex patterns with non‐significant changes for C380, substantial reduction for C386, and non‐significant oxidation for C398, indicating that circadian and light/dark responses involve different cysteine modification patterns.

Overall, the computational analyses (Figures 1, 2, 3), combined with the experimental redox proteomics data (Figure 3), highlight three key findings: first, two distal regions within OpcA (loop 160–174 and Region IV 377–404) exhibit flexibility that is highly affected by thiol PTMs. Both of them are located at the grooves of subunits A, B, and C of the tetrameric G6PDH complex; second, these five cysteine sites in these regions (C162, C174, C380, C386, and C398) tend to be oxidized under dark conditions; and third, the two disulfide bonds in these regions of OpcA, corresponding to C162‐C174 and C380‐C386, play a pivotal role in the activity of G6PDH in different cyanobacteria (Doello et al., 2024), while glutathionylation at C398 represents one of the most energetically favorable PTMs. Given that these three PTMs occur near the interfacial region between the OpcA and G6PDH in their complex, they likely significantly affect the structure and function of G6PDH under light variation.

### 2.3 Thiol PTMs of OpcA in proximity to the G6PDH complex potentially regulate substrate access through allosteric gate modulation

Based on our experimental redox data, as well as a recent cryo‐EM study of other cyanobacteria (Doello et al., 2024), we focused on the cysteine residues in the two interfacial regions of OpcA at the interface with G6PDH (C162, C174, C380, C386, and C398). We constructed and simulated two different OpcA–G6PDH complex systems: one with completely reduced cysteines without any PTMs or disulfide bonds, and another with two disulfide bonds (C162–C174 and C380–C386) and one glutathionylated PTM at C398. We calculated relative binding free energies (RBFE) to test our hypothesis regarding the impact of cysteine PTMs in OpcA on the structure and function of the G6PDH complex. Hereafter, we refer to these systems as the reduced and PTMed complexes, respectively.

**FIGURE 4:**
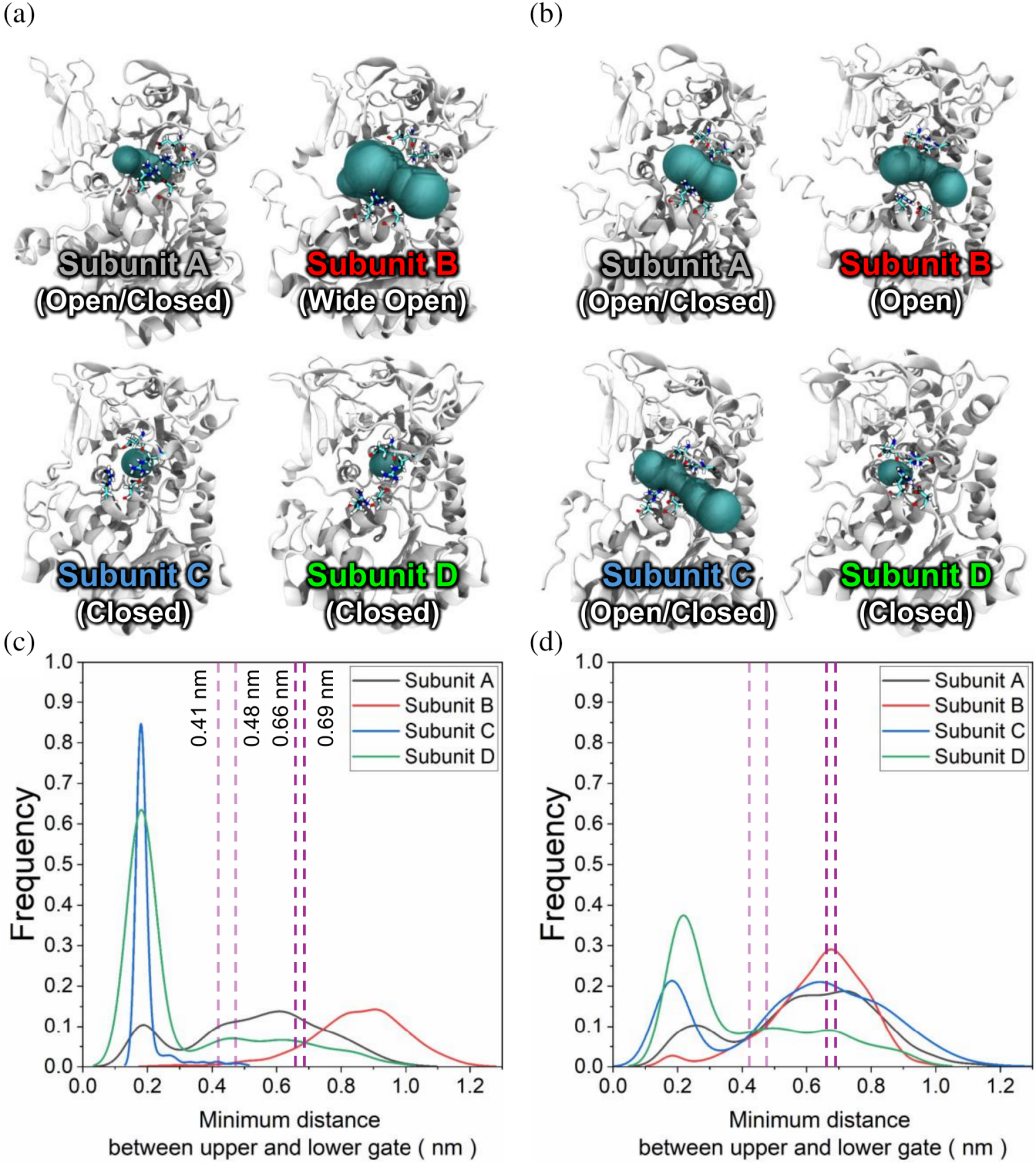
Representative snapshots of each subunit in the G6PDH complex in the (a) reduced and (b) oxidized cases with PTMs. The void area at the active site pocket is highlighted by cyan regions. Relative frequencies of the gate configurations in the (c) reduced and (d) oxidized cases with PTMs. Distributions of subunits A, B, C, and D are highlighted in black, red, blue, and green, respectively. Purple vertical dashed lines represent the distances between the upper and lower parts of the gate for G6PDH protein complexes from different cyanobacteria.

Our simulations demonstrated that the PTMed complex showed an increase of ~18.5 kcal/mol in the OpcA–G6PDH interfacial non‐bonded interaction energy (shifting toward less negative values), corresponding to a weakening of binding affinity after PTM (Figure S4), while overall structural flexibility remained similar between the two states (Figure S5). This indicates that cysteine PTMs in the critical regions of OpcA reduce interfacial binding strength without substantially altering the global conformational flexibility of the complex. Our simulation results also showed that PTMs significantly influence the structures and dynamics of a pocket near the active site of G6PDH (Figure 4). This pocket (in cyan) appears to function as a molecular gate, controlling the ingress of substrates or cofactors and the egress of reaction products. In the reduced complex, the gate adopts either a closed or wide‐open conformation (Figure 4a), whereas PTMs modulate the gate conformations, resulting in more stable, open conformations upon PTM formation (Figure 4b).

By analyzing the distribution of the distance between the upper and lower sides of the gate in cryo‐EM‐based 3D structures of the G6PDH complex from different cyanobacteria, which range from 0.4 to 0.7 nm (Figure 4c,d), we categorized the G6PDH structures into three types of distributions: the distribution of the “Open” conformations, where the peak of the profiles is located around 0.7 nm; the distribution of the “Closed” conformations, where the peak is situated at distances less than 0.4 nm; and the distribution of “Wide‐open” conformations, where the peak position exceeds 0.7 nm (Figure 4). In the reduced complex, two of the four G6PDH subunits showed a “closed” gate, leading to reduced inner space near the catalytic cavity; one subunit showed alternating “open/closed” gate conformations with limited void volume near the active site; and one adopted a “wide‐open” conformation with a large space in the cavity. However, when OpcA was oxidized with thiol PTMs, three out of four G6PDH subunits showed either “open” or “open/closed” conformations, and the only one subunit exhibited a “closed” conformation. Subunit D, which in both the PTMed and reduced cases showed “closed” gate conformations, is the subunit that lacks close contacts with OpcA when assembled (Figure 2c), suggesting that interactions with OpcA indeed influence the complex's structure.

### 2.4 Thiol PTMs of OpcA modulate gate conformations and hydrogen bond networks within the G6PDH active site

**FIGURE 5:**
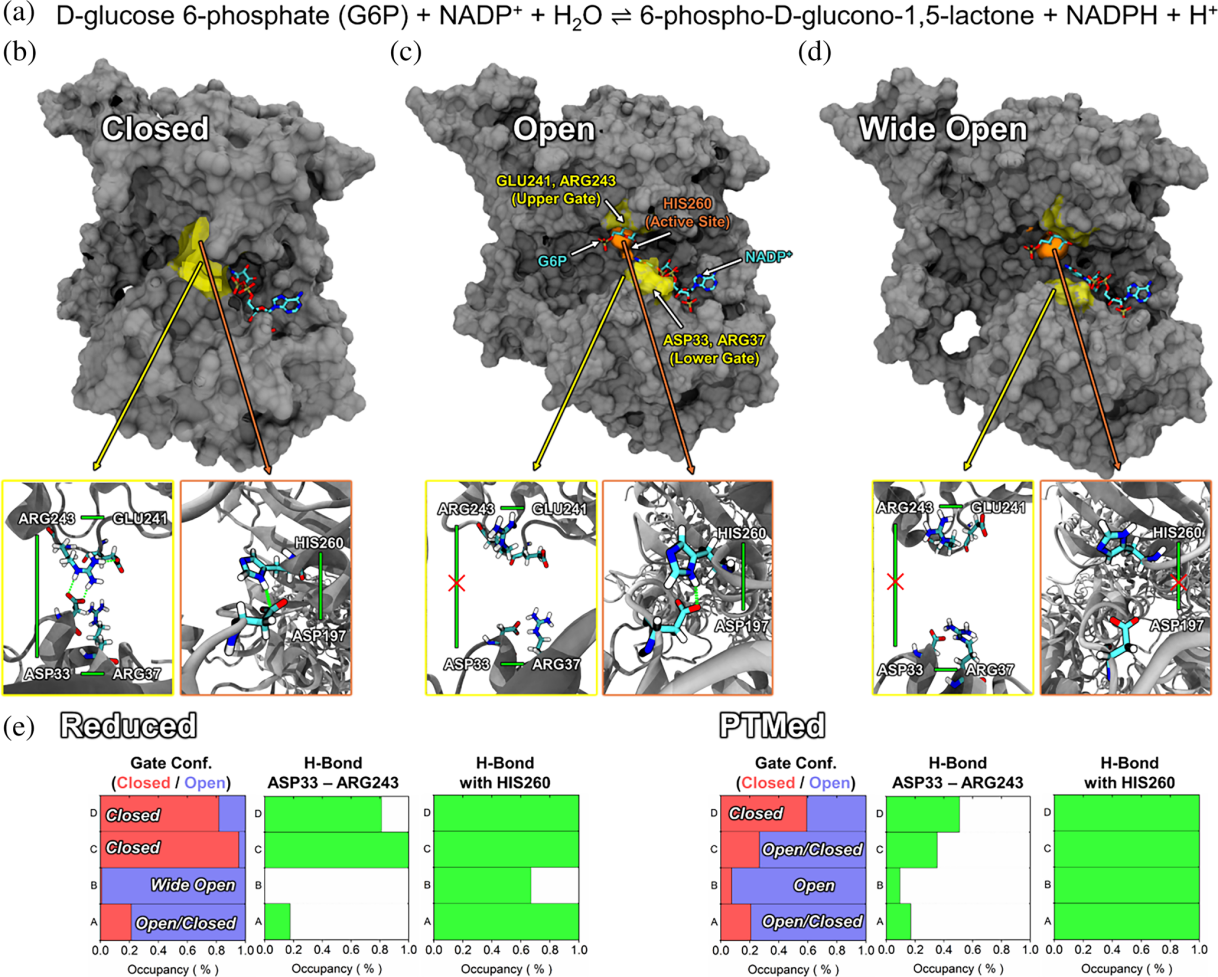
(a) The proposed reaction occurring at the active site of G6PDH. Representative snapshots of the G6PDH protein with three different gate conformations: (b) closed, (c) open, and (d) wide‐open. The substrate and cofactor, G6P and NADP^+^, were taken from the human G6PDH and docked into our system to better understand how reactants can bind to the active sites. (e) Left: Occupancy of the gate in the open (blue) and closed (red) states for both the reduced and PTMed complexes; Middle: Occupancy of the hydrogen bond between ASP33 and ARG243; Right: Occupancy of the hydrogen bond involving HIS260.

The upper and lower regions of the gate are composed of pairs of charged amino acids, ASP33 and ARG37, for the lower part, and GLU241 and ARG243 for the upper part (Figures S1 and 5). Our simulations showed that the open and closed configurations arise from hydrogen bonding between ASP33 and ARG243. In the closed state, these two amino acids move closer and tend to form hydrogen bonds (Figure 5b,e), whereas in the open or wide‐open states, they are positioned farther apart, resulting in minimal or no hydrogen bonding (Figure 5c–e). As shown in Figure 5e, the occupancy of the gate open and closed states in both reduced and PTMed complexes exhibits an almost exact correspondence with the hydrogen‐bond occupancy between the two residues. This strong correlation suggests that the hydrogen bond formed between these residues functions as a key structural determinant for the gate conformation. Interestingly, when the gate is wide‐open, the local structure near the active site is disrupted, leading to the loss of crucial hydrogen bonds between active site residues, including ASP197 and HIS260 (Figures 5d, e and S1). Similar gate mechanisms that directly affect enzyme activity and reaction rates have been reported across various biocatalytic systems (Gora et al., 2013; Kokkonen et al., 2018). For example, recent combined experimental and computational studies have shown that enzyme activity and reaction rates are optimized when the ingress and egress gates remain in the “open” conformation. Conversely, enzyme activity and catalytic rates can be significantly reduced when the gate is “closed” or “wide‐open” (Kim et al., 2014; Kim et al., 2016). This indicates that maintaining a stable open conformation ensures optimal enzyme activity, while activity can be significantly diminished in the closed state due to restricted substrate or product ingress and egress, or in the wide‐open state due to unstable interactions between active site residues. Given that the residues involved in the gate configurations and the active site are conserved (Figure S3), the proposed gate mechanism could potentially play a similar role in G6PDH complexes across different organisms (e.g., humans), where substrate access may be regulated through analogous structural features even in the absence of OpcA.

### 2.5 Allosteric regulation by PTM emerges from redistribution of collective dynamics

**FIGURE 6:**
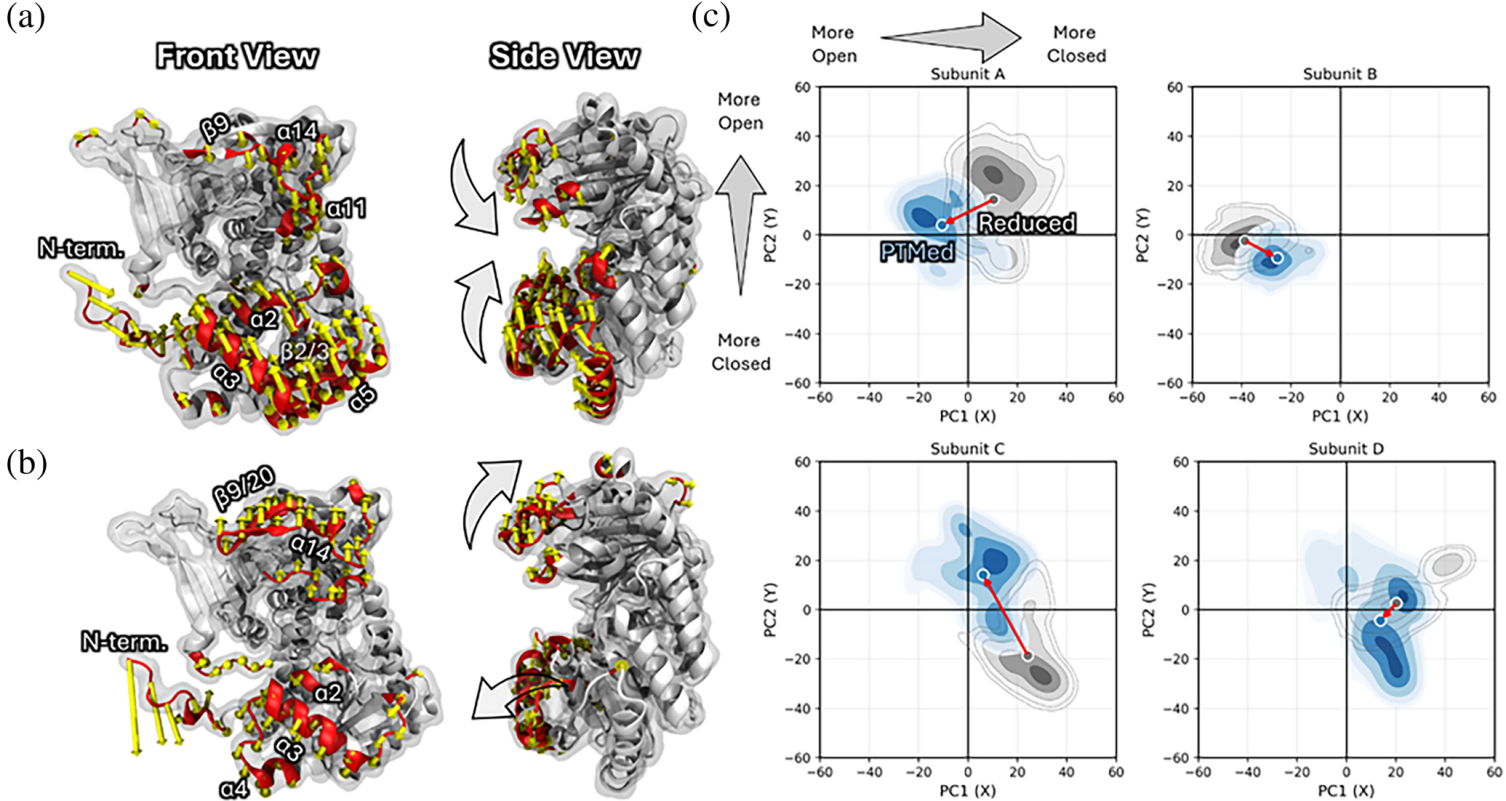
(a) PC1 and (b) PC2 displacement vectors mapped onto a representative G6PDH subunit. Yellow arrows, with red‐highlighted residues, labeled by secondary structure, represent the cumulative top 50% eigenvector contributions; gray arrows indicate the direction of motion. (c) Projection of reduced (gray) and PTMed (blue) trajectories onto PC1–PC2 space. Red arrows indicate centroid shifts.

To determine how PTMs of OpcA reshape collective gate dynamics in the G6PDH complex, we performed principal component analysis (PCA) on combined reduced and PTMed trajectories projected onto a common eigenspace (Figure 6). PC1 (36.2%) and PC2 (15.6%) together account for over 50% of the total positional variance and capture the dominant collective motions of the G6PDH tetramer. Eigenvector mapping shows that more than 50% of the mode amplitude is concentrated in secondary structures surrounding the catalytic cleft, including α‐helices (α2, 3, 5, 11, 14) and β‐strands (β2, 3, 9, 20), indicating that both principal components are intrinsically coupled to gate mechanics (Figure 6). PC1 describes a hinge‐like displacement modulating open and closed conformations of the cleft, whereas PC2 captures asymmetric breathing‐like motions of the same structural elements; projections confirm that both modes contribute to gate opening and closing (Figures 6 and S6). Rather than generating a new collective mode, PTM redistributes the weights, or the coefficients of PC1 and PC2 in a subunit‐dependent manner (Figures 6 and S6).

Among the four subunits, Subunit A of the G6PDH complex shows the most displacement in PC1 between reduced and PTMed OpcA, but it remains near the central region of PC space, indicating no qualitative change in its open/closed motions. Subunit B shifts mainly along PC1 toward more positive values, consistent with relaxation from a wide‐open to a moderately open state. Subunit C also exhibits a pronounced PC2‐directed centroid shift toward more open conformations, consistent with the shift from a predominantly closed to a mixed open/closed state. In contrast, Subunit D remains largely closed in both states, maintaining centroid positions within the closed region of PC space. Overall, the PTMs of OpcA reorganize the conformational landscape of the binary complex, and that governs the collective gate opening and closing of G6PDHs.

To determine how the PTM‐induced redistribution of collective modes manifests at the system level, we next analyzed full residue–residue correlation matrices (Figure S7). The correlation coefficient C_ij_ quantifies dynamic coupling between residues: positive values (*C*_*ij*_ >0.2) indicate correlated motion, where residues move in the same direction, whereas negative values (*C*_*ij*_ < −0.2) indicate anti‐correlated motion, where residues move in opposite directions. Residue pairs with −0.2 < *C*_*ij*_ <0.2 were considered weakly correlated or uncorrelated. Comparison of the reduced and PTMed systems reveals a broad reorganization of correlated and anti‐correlated interactions across the G6PDH tetramer and OpcA. In the PTMed system, clusters of strongly correlated motion become less dominant, while anti‐correlated regions extend across multiple subunits. Rather than indicating localized perturbations, this redistribution reflects a restructuring of long‐range dynamical coordination within the complex, consistent with the PTM‐dependent reshaping of collective gate motions observed in PCA.

**FIGURE 7:**
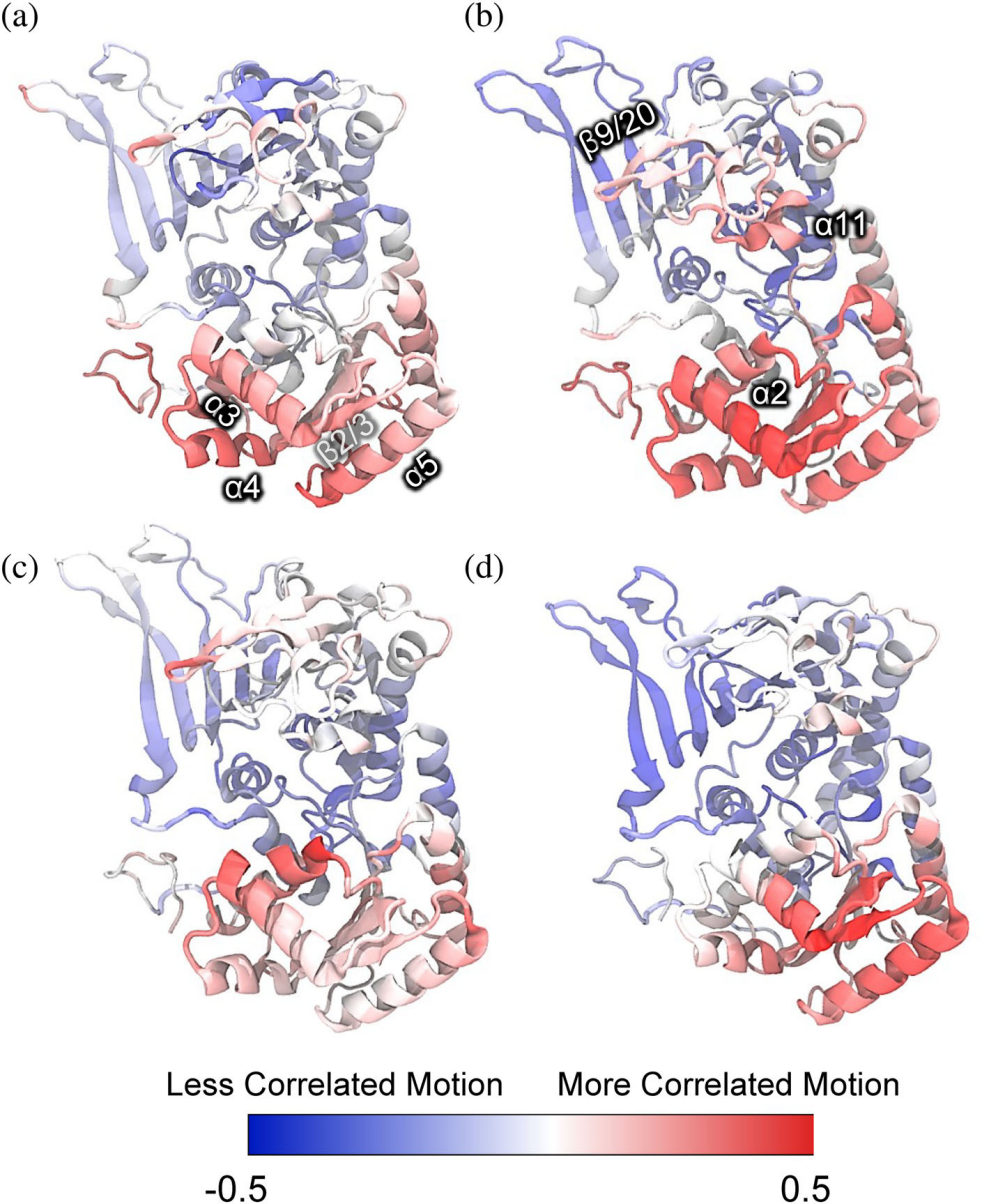
(a–d) Residue‐wise correlation redistribution score mapped onto G6PDH subunits A–D. For each residue, the score represents the PTM‐induced change in the balance of correlated and anti‐correlated interactions relative to the remainder of the complex (G6PDH tetramer + OpcA). Red indicates net enrichment of correlated interactions, whereas blue indicates relative enrichment of anti‐correlated interactions upon PTM.

While the full correlation patterns are complex (Figure S7), they consistently suggest PTM‐dependent reweighting of how gate‐associated residues dynamically couple to the rest of the protein. To quantify this effect, we computed residue‐wise redistribution scores which measure, for each G6PDH residue, the PTM‐induced redistribution of its dynamic coupling with all other residues in the system (Figure 7). Specifically, for each residue, we computed the fraction of strongly correlated (*C*_*ij*_ >0.2) and anti‐correlated (*C*_*ij*_ < −0.2) interactions with all other residues in the complex in both the reduced and PTMed systems and defined the PTM effect as the difference between these two states. The resulting score reports the net enrichment or depletion of correlated versus anti‐correlated interactions upon PTM, thereby capturing how each residue's global dynamical connectivity is re‐weighted within the complex (Figure 7). Our results show that residues near the catalytic cleft, particularly α2, α3, α4, and α5 and adjacent structural elements, consistently display increased correlated interactions in the PTMed system across all four G6PDH subunits. This enrichment of correlated interactions is consistent with enhanced concerted coordination among gate‐forming elements, whereas many peripheral regions exhibit reduced correlated motion. Notably, these same structural elements correspond to the dominant eigenvector contributions identified in PCA (Figure 6), directly linking strengthened gate‐centered coordination to the redistribution of conformational sampling along PC1 and PC2.

Together, these results indicate that PTM‐driven redistribution of collective modes arises from a selective reorganization of dynamical couplings within the complex. The redistribution of correlated and anti‐correlated interactions provides a mechanistic link between long‐range coordination and the redistribution of conformational sampling observed in PCA, thereby modulating individual gate opening and closing behavior of each subunit.

### 2.6 Residue‐level interaction patterns are associated with PTM‐dependent gate regulation

To complement the dynamics‐based analyses, we examined residue–residue non‐bonded interactions between OpcA and G6PDH to identify representative interaction patterns associated with PTM‐dependent gate conformations (Figure S8). These interactions are not intended to define unique allosteric pathways, but rather to illustrate residue‐level coupling patterns accompanying the redistribution of collective dynamics. Across subunits and conformational states, several G6PDH residues dynamically couple to three key regions of OpcA and participate in interactions involving gate‐associated residues. As shown in Figure S8, in open/closed states, LYS38 and ASN259 frequently engage in electrostatic interactions with gate residues under OpcA influence. As conformations shift toward more closed states, GLN87 contributes more prominently, while the apparent influence of OpcA becomes reduced. In contrast, in open and wide‐open conformations, long‐range coupling shifts toward residues such as ARG323 and TYR309, with more limited OpcA involvement. Importantly, these key residues are located within secondary structural elements that were previously identified as key regions (e.g., α2, α14, β9 or adjacent loops) exhibiting strong dynamic coupling changes. Thus, the residue‐level interaction patterns observed here are not isolated phenomena, but rather local manifestations of the global PTM‐induced reorganization of collective gate dynamics. This network‐level interpretation is consistent with recent studies in other protein systems employing elastic network models, which support a view of allostery arising from distributed dynamical coupling across the protein structure (Duman et al., 2025). Overall, these residue‐level interaction patterns are consistent with the PTM‐dependent redistribution of dynamical coordination described above and provide illustrative examples of how global reorganization of collective modes may manifest locally at the gate interface.

## 3 DISCUSSION

### 3.1 Thiol PTMs regulate molecular assembly, energy production, and metabolic flux

In cyanobacteria, G6PDH is not directly redox‐sensitive, and its activity is modulated by thiol PTMs of the OpcA protein that binds to the G6PDH complex (Doello et al., 2024). Experimental studies have demonstrated that the activity of G6PDH is drastically reduced in the absence of OpcA (Hagen & Meeks, 2001). Notably, the oxidized form of OpcA can lower the *K*_*m*_ for G6P, highlighting the importance of the redox state of OpcA for the G6PDH enzyme function and cellular capacity to generate NADPH when reducing power is needed (Mihara et al., 2018). More specifically, a recent cryo‐EM study revealed two crucial intramolecular disulfide bonds of OpcA in the cyanobacterial strain *Synechocystis* sp., which play an essential role in the function of G6PDH proteins, acting as an allosteric activator (Doello et al., 2024). Our simulation results, combined with redox proteomics, also demonstrate that these two disulfide bonds of OpcA influence the structural dynamics of the G6PDH‐OpcA complex as well as the G6PDH enzyme activity. In addition, glutathionylated Cys398 also affects complex structure, dynamics, and function, which was not observed in previous studies (Figures 3, 4, 5).

Using the PTM‐Psi tool we developed (Mejia‐Rodriguez et al., 2023), we discovered for the first time that the activity of the G6PDH‐OpcA complex in the OPPP is strongly linked to structural changes in the active site of G6PDH subunits induced by thiol PTMs (Figures 4, 5, and S5). When OpcA is reduced—where two disulfide bonds and glutathionylated cysteine are not present—the active site pocket in the four subunits of the G6PDH complex differentially exhibits either “closed” or “wide‐open” configurations, which are not optimal for substrate binding or enzyme reactions. In contrast, upon oxidation, the distribution of the gate distances aligns for most subunits of G6PDH, with the exception of Subunit D that lacks direct interactions with OpcA. The active site pockets are predominantly maintained in optimal open configurations. PTM at OpcA propagates the collective dynamics across the OpcA–G6PDH complex, beyond local structural changes at the gate and active site (Figures 6, 7, and S8). Experimentally, redox proteome (Figure 3) provided evidence on the differential expression of PTMs particularly on the cysteines of OpcA at the G6PDH binding site, Region IV. We speculate that PTMs tune the conformational landscapes of individual G6PDH subunits toward functionally relevant configurations according to environmental gradients.

### 3.2 Hierarchical regulation of cellular reducing power production and carbon flux through dual temporal mechanisms and distinctive phenotypes under experimental gradients

The primary biological function of the OpcA‐G6PDH system is maintaining cellular reducing power balance through NADPH generation when photosynthetic electron transport is unavailable or insufficient. The formation and reduction of cysteine‐based PTMs occur on the sub‐second‐to‐minute time scale—several orders of magnitude faster than transcriptional or translational control (Doello et al., 2024; Johnson et al., 2025; Johnson et al., 2026; Li et al., 2023). Within this kinetic window, OpcA operates as a molecular “rheostat” that couples the cellular redox state to immediate NADPH production through the OPPP. When light intensity drops or reactive oxygen species accumulate, thiol oxidants promote rapid intramolecular disulfide formation. The oxidized form of OpcA engages G6PDH with high affinity, boosting NADPH production to meet urgent cellular reducing power demands. The additional reducing power is immediately channeled to (1) antioxidant systems such as peroxiredoxins and glutathione reductase, and (2) anabolic reactions that rely on NADPH (e.g., CO_2_ fixation and glycogen synthesis). Conversely, reduction of the disulfide by thioredoxin, itself fueled by photosynthetic ferredoxin, renders the OpcA‐G6PDH inactive within seconds after light returns. This “on–off” cycle prevents futile NADPH overproduction when photosynthesis can adequately supply reducing power and redirects glucose‐6‐phosphate back into glycolysis and the CBB cycle. Within this cycle, the molecular gate conformation, regulated by OpcA, could serve as a key mechanism for controlling this dual temporal process (Johnson et al., 2025).

Complementing this rapid PTM‐based regulation, we observed a slower regulatory method through substantial changes in the abundance of OpcA and G6PDH proteins when comparing diel cycle‐adapted cells at noon (cells rely on photosynthesis as the source of reducing power) to cells at subjective dusk (cells prepare for light being unavailable). Indeed, our previously published analysis of the *S. elongatus* transcriptome under diel conditions showed that both genes, *opcA* and *zwf*, encoding the G6PDH protein, were among the most differentially expressed genes between dusk (8:00 p.m.) and noon (12:00 p.m.) time points under identical light conditions (Gilliam et al., 2025). Therefore, this slower, abundance‐based control mechanism has a dramatic impact on cellular bioproduction function while operating on an hourly timescale. Importantly, our redox proteomics analysis showed that light perturbation and circadian transitions selectively modify distinct cysteine pairs in OpcA, indicating that environmental redox regimes generate site‐specific PTM patterns rather than uniform oxidation. These environmentally responsive thiol modifications provide a direct mechanistic link between external light‐driven cues and the redistribution of collective gate dynamics described above. Because the PTM switching process is both reversible and localized, it enables a layered regulatory hierarchy: an ultrafast layer (ms–s), where cysteine redox toggles OpcA/G6PDH activity and, by extension, NADPH supply; and a slow layer (h–d) where changes in gene expression alter absolute enzyme abundance, locking in the metabolic state established by earlier redox cues. Moreover, it is possible that an intermediate layer (min–h) exists, where metabolite feedback (e.g., accumulation of 6‐phosphogluconate, ATP/ADP ratio) fine‐tunes flux partitioning between the OPPP and glycolysis/CBB (Esposito, 2016; Jiang et al., 2022; Preiser et al., 2019; Xu et al., 2024).

Such temporal partitioning ensures that cyanobacteria can respond to rapid environmental fluctuations through immediate NADPH mobilization while simultaneously adapting their proteome to sustained environmental changes. From a systems perspective, OpcA therefore acts as an integrator that coordinates reducing power homeostasis with carbon, nitrogen, and redox metabolism in real time, providing a paradigm for how single redox switches can orchestrate global pathway crosstalk with the primary goal of maintaining cellular reducing power balance. Similarly, a spatiotemporal whole‐cell model of *Prochlorococcus marinus* demonstrated that redox PTM‐mediated assembly of the CP12‐Gap‐Prk dark complex regulates CBB cycle flux under light disturbance, reinforcing the broader role of thiol redox switches in cyanobacterial carbon metabolism (Johnson et al., 2026).

### 3.3 PTM‐Psi integrated with redox proteomics provides a powerful approach for detecting cellular states by linking molecular details to systems modeling

High‐coverage redox proteomics now delivers quantitative site‐occupancy data for thousands of cysteine residues, but converting these datasets into mechanistic insight remains challenging. PTM‐Psi closes this gap by providing physics‐based structural context for each modified site. The workflow is conceptually straightforward. Looking forward, integrating PTM‐Psi/redox‐proteomics data with machine‐learning frameworks and time‐resolved proteomics will enable real‐time tracking of cellular states, guiding interventions ranging from developing stress‐resilient and high‐productivity cyanobacterial strains with optimized reducing power management to therapeutic modulation of redox‐sensitive enzymes in human disease.

## 4 CONCLUSIONS

This study shows that thiol post‐translational modifications (PTMs) act as allosteric switches that regulate cyanobacterial reducing power balance and carbon metabolism by structurally modulating the OpcA–G6PDH complex. Using an integrative PTM‐Psi/redox proteomics approach, we identify mechanisms by which redox‐sensitive cysteine modifications likely enable the rapid metabolic response of *Synechococcus elongatus* PCC 7942 to fluctuating environmental conditions.

At the molecular level, our simulations indicate that specific cysteine PTMs in OpcA, formation of C162–C174 and C380–C386 disulfide bonds and C398 glutathionylation, serve as conformational switches that reshape the conformational landscape of G6PDH subunits. Rather than enforcing a single static conformation, these PTMs bias G6PDH toward catalytically favorable states in a manner that depends on environmental conditions. They promote optimal “open” or “open/closed” gate conformations in three of four G6PDH subunits while potentially preserving critical hydrogen‐bond networks (ASP33–ARG243) required for active‐site integrity. The predicted increase in OpcA–G6PDH interfacial energy upon PTM formation indicates weakened interfacial interactions, suggesting that redox‐dependent regulation arises from PTM‐induced reorganization of structural and dynamical couplings within the complex, rather than tighter complex formation. Beyond local structural changes, our results support a model in which PTMs regulate G6PDH activity by reorganizing collective dynamical coupling across the complex. This dynamical bias provides a mechanistic link between redox sensing at OpcA and G6PDH regulation without requiring a single, rigid allosteric pathway. These findings demonstrate that PTM‐level regulation provides a critical control layer from genotypes to phenotypes that enables cyanobacteria to rapidly adapt to environmental fluctuations through precise metabolic fine‐tuning.

## 5 MATERIALS AND METHODS

### 5.1 Global and redox proteomics experiments

Global and redox proteome experiments were performed to investigate the redox state of cysteine residues in the OpcA protein using the *S. elongatus* PCC 7942 cscB/SPS strain. This strain was selected as a bioproduction‐relevant model system, and the experiment was designed to mimic physiological transitions where OpcA is involved in modulating cellular reducing power generation and carbon flux through activation of the OPPP. We established two distinct *S. elongatus* cultures: one adapted to continuous light and a second diel culture that was adapted to a 14:10 h light–dark cycle for approximately 6 months. Both cultures were cultivated under ~200 μmol m^−2^ s^−1^ photon flux density at 29 ± 2°C and supplied with 2% CO_2_. The continuous light culture was grown in a modified BG‐11 medium containing 0.09 g L^−1^ Yeast Nitrogen Base without amino acids and ammonium sulfate (H26271.36, Thermo Fisher Scientific Inc., USA), 0.264 g L^−1^ of (NH_4_)_2_SO_4_ (J64180.A1, Thermo Fisher Scientific Inc., USA), and 0.174 g L^−1^ of K_2_HPO_4_, (60,356, Sigma‐Aldrich, USA). Cultures were maintained in the exponential growth phase through regular dilution with fresh medium to maintain an OD_750_ of approximately 0.2.

To investigate how light–dark transitions and day‐night dynamics affect the redox state of the OpcA protein, we performed three complementary experiments. The first two experiments—one with *S. elongatus* cultures at high cell density (OD_750_ 1.0) and another at low cell density (OD_750_ 0.08)—measured changes in the redox state of OpcA after transitioning from light to dark conditions. The third experiment measured changes in the redox state of OpcA in diel‐synchronized cultures at different points of the circadian cycle, specifically at dusk (8:00 p.m.) versus noon (12:00 p.m.) under identical light conditions.

For the light‐to‐dark transition experiments, cultures were cultivated under continuous light from OD_750_ 0.15 to OD_750_ 1.0 (late mid‐exponential phase) over 4 days. After reaching the desired optical density, half of the culture was divided into individual culture flasks that served as experimental replicates for light‐to‐dark transition under high‐density conditions. The remaining half was diluted from OD_750_ 1.0 to 0.08 by using filter‐sterilized spent medium from the same cell culture. Both dense and dilute cultures were then exposed to the same light intensity for 0.5 h, and light condition samples were collected for both densities. Importantly, individual cells in the dilute culture (OD_750_ 0.08) received much higher light irradiance due to reduced self‐shading compared to cells in the dense culture (OD_750_ 1.0). Subsequently, the cultures were transferred to darkness for 2 h, and dark condition samples were collected.

For the circadian cycle comparison experiment, we cultured two groups of diel cycle‐adapted cells and harvested one group at 12:00 p.m. (noon) and another group at 8:00 p.m. (dusk). A detailed description of this experiment was reported earlier (Gilliam et al., 2025). For each biological replicate, 100 mL of culture was divided into two 50 mL aliquots and centrifuged at 4700*g* for 5 min at 4°C. The supernatant was decanted, and cell pellets were flash frozen in liquid nitrogen and stored at −80°C. Dark treatment samples were maintained in darkness during all processing steps. Samples were then processed as described previously (Guo et al. 2014c). Briefly, cell pellets were treated with 10% cold trichloroacetic acid on ice, then incubated in 250 mM HEPES (pH 7.5) containing 8 M urea, 10 mM ethylenediaminetetraacetic acid (EDTA), 0.5% sodium dodecyl sulfate (SDS), and 100 mM N‐ethylmaleimide (NEM) for 2 h, then lysed by bead beating. The extracted protein was precipitated and washed with cold acetone, then reduced with dithiothreitol. The reduced protein was further processed with a resin‐assisted capture (RAC) protocol to selectively enrich proteins with oxidized cysteine residues (Guo et al. 2014b). A detailed experimental procedure for the redox proteome was described earlier (Gaffrey et al., 2021).

Simultaneously, an aliquot was taken from each protein sample before resin‐assisted capture enrichment to quantify global protein expression levels. Isobaric labeling was used for quantification. The enriched samples and global protein samples were analyzed by a nanoAcquity UPLC system (Waters) coupled to a Q‐Exactive HF‐X Orbitrap mass spectrometer (Thermo Scientific, San Jose, CA) with a 120‐min liquid chromatography (LC) gradient. Full mass spectrometry (MS) scans were acquired in the range of m/z = 400–1800. MS/MS was performed in data‐dependent mode. The parent ion tolerance was 20 ppm. LC–MS/MS raw data were searched against the *S. elongatus* PCC 7942 UniProt database (downloaded March 8, 2022) using MS‐GF+ (Guo et al. 2014b; Kim & Pevzner, 2014). A detailed experimental procedure for this process was described previously (Guo et al. 2014b).

### 5.2 Computational study using PTM‐Psi

**FIGURE 8:**
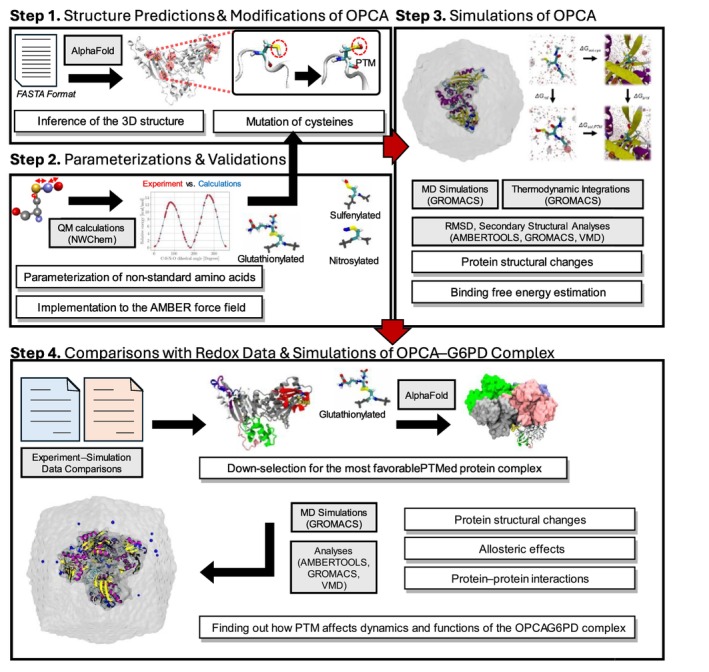
A computational workflow depicting the sequence of steps in this study. Steps 1 to 3 illustrate the automated steps of this study using the PTM‐Psi package: From protein structure inference and parameterizations for the simulation to actual MD simulations of the OpcA protein using PTM‐Psi. Step 4 is a further step of this research, including comparisons between experimental and computational data, down‐selection of representative cases for simulations of the OpcA‐G6PDH complex, and relevant simulations and analyses.

We used PTM‐Psi to study the impact of PTM on the structure and function of OpcA (Mejia‐Rodriguez et al., 2023; Samantray et al., 2025). A workflow of PTM‐Psi, a Python package to facilitate the computational investigation of post‐translational modification on protein structures and their impacts on dynamics and functions, is provided in Figure 8. PTM‐Psi is a user package that we previously developed to automate the various computational tasks, including building systems with multiple combinations of PTM on target proteins, implementing and validating force fields using quantum mechanics (QM) approaches for the non‐standard PTMed amino acids, performing MD simulations, and analyzing simulation trajectories.

### 5.3 MD simulations of OpcA with PTMs

The PTM‐Psi package provides a streamlined, end‐to‐end automated workflow from a protein sequence to trajectory analysis. Here we break down each step for clarity. First, we obtained a primary protein (FASTA) sequence of OpcA from *S. elongatus* strain PCC 7942 (hereafter, *S. elongatus*) from UniProt (ID:Q54709, OpcA_SYNE7) and used AlphaFold 2 (Jumper et al., 2021) to infer its 3D structure (Figure S1). The predicted structure was compared with the experimentally verified OpcA from a different cyanobacterium, *Synechocystis* sp. PCC 6803 (Doello et al., 2024). using the MM‐align tool to get the TM‐score for assessing protein similarities (Mukherjee & Zhang, 2009). Two structures with TM‐scores between 0.5 and 1.0 have considerably high structural similarities (Zhang & Skolnick, 2004) and, as illustrated in Figure S2, the predicted OpcA structure showed good alignment with the cryo‐EM‐based structure (Doello et al., 2024). Using PTM‐Psi, all cysteine residues were considered PTM sites and modified to three different types of PTM: sulfenylated, nitrosylated, and glutathionylated. The force field parameters of these non‐standard amino acids, including both bonded and non‐bonded terms, were carefully parameterized and validated using the QM approach. (Mejia‐Rodriguez et al., 2023). These parameters shown in Tables S1 and S2 were integrated with the PTM‐Psi package, extending the capabilities of the AMBER99SB force field (Hornak et al., 2006). Based on the number of cysteine sites and three different PTM types, we built 34 different systems (11 cysteines × 3 PTM types + 1 completely reduced OpcA) and created three replicas per system for improved sampling from the MD simulations. A total of 102 unique cases were then solvated in a truncated dodecahedron periodic box filled with TIP3P water molecules (Jorgensen et al., 1983) with monovalent ions based on Joung and Cheatham ion parameters (Joung & Cheatham, 2008) to neutralize the charge. GROMACS 2024.3 (Abraham et al., 2024) was used for the MD simulations. The equilibrations began with solvent minimization while keeping the protein atoms restrained at 1000 kJ mol^−1^·nm^−2^. Additional multistep minimizations were subsequently performed, where harmonic restraints on the protein atoms were gradually reduced from 500 to 200, 100, 10, 5, and 1 kJ mol^−1^ nm^−2^. Then, the system was equilibrated at 300 K for 500 ps in the constant‐temperature, constant‐volume (NVT) ensemble using velocity rescaling with a stochastic term thermostat (Bussi et al., 2007), followed by 500 ps equilibration at 1 bar and 300 K under a constant‐temperature, constant‐pressure (NPT) ensemble using the stochastic cell rescaling (C‐rescale) method for pressure control (Bernetti & Bussi, 2020). The final minimization was carried out without harmonic restraints. Afterward, the system was re‐equilibrated without any restraints using the same procedures for the NVT and NPT ensembles. The systems were then propagated for 100 ns at 300 K and 1 bar using the Parrinello–Rahman pressure coupling method (Parrinello & Rahman, 1981) along with the particle mesh Ewald method for long‐range electrostatic interactions (Darden et al., 1993), and employing the linear constraints solver (LINCS) (Hess, 2008; Hess et al., 1997) to constrain all bonds to hydrogen atoms. All simulations adopted a timestep of 2 fs.

Subsequently, we performed thermodynamic integrations (TI) of all distinct systems to compute the free energy cost associated with transforming a protonated cysteine (CYS) state to its sulfenylated, nitrosylated, or glutathionylated form. In the TI approach, non‐bonded and bonded interactions were changed via a two‐step method where electrostatic contributions were decoupled first, followed by van der Waals, masses, and bonded terms. These terms were gradually decoupled by increasing the coupling parameter (λ) from 0.00 to 1.00 across 13 discrete windows (λ = 0.00, 0.05, 0.10, 0.20, …, 0.90, 0.95, 1.00), for each decoupling step. Thus, a total of twenty‐six 1 ns trajectories were employed for the calculation. TIs were also performed for the same transitions in TIP3P water (Jorgensen et al., 1983), which were used as reference values to obtain the relative binding free energies (RBFE). Free energy differences were obtained using Bennett's acceptance ratio method (Bennett, 1976) as implemented in the g_bar module of the GROMACS simulation package (Abraham et al., 2024). The RBFEs were calculated as the differences between the free energy costs observed in the protein systems and those in water. This alchemical process was incorporated in PTM‐Psi, with further details provided in our previous papers (Mejia‐Rodriguez et al., 2023; Samantray et al., 2025).

### 5.4 MD simulations of the reduced and PTMed OpcA‐G6PDH binary complexes

For simulating the OpcA‐G6PDH binary complex, a protein sequence of G6PDH from the same strain, *S. elongatus*, was first obtained from the UniProt database, P29686, G6PDH_SYNE7 (Figure S1). We used AlphaFold 3 (Abramson et al., 2024) to predict the hetero‐oligomeric structure composed of a monomer of OpcA and a tetramer of G6PDH proteins. For the prediction, five models were generated and ranked according to predicted local difference distance test (LDDT) and predicted aligned error (PAE) scores. The top‐ranked model was selected as the starting structure for molecular dynamics simulations. To assess conformational variability, the prediction was repeated two additional times using different random seeds, producing a total of 25 models. The RMSD among these models was 0.0660 ± 0.0197 nm, indicating that the model selected for the MD simulations is marginally different from other predicted ones. This supports the suitability of our selected model as a reasonable starting structure. However, while AlphaFold 3 provides high‐confidence predictions, these structures could represent a dominant conformational state; thus, the simulations probe PTM‐dependent modulation of dynamics around this reference structure rather than full conformational sampling. MM‐align (Mukherjee & Zhang, 2009) was used to assess the structural similarity between predicted and cryo‐EM‐based complex structures, demonstrating good structural similarity (Figure S2). Based on our computational RBFE results, along with a previous study (Doello et al., 2024) and experimental redox proteome data, we downselected cysteine sites that tend to be PTMed under dark conditions and created two distinct complexes for further simulations: one is a completely reduced complex where no disulfide bonds or thiol PTMs are formed, the other is a PTMed complex with two disulfide bonds, C162‐C174 and C380‐C386, as well as glutathionylated C398. The aforementioned procedures were applied for the minimization and equilibration of these two OpcA‐G6PDH binary complexes. To examine PTM‐induced structural changes, we conducted extended production runs. For both the reduced and PTMed OpcA‐G6PDH binary complexes, three independent replicas were prepared, and each system was simulated for 500 ns, yielding a total of 1500 ns of sampling per condition. Simulation lengths were chosen to compare PTM‐dependent dynamical trends across multiple replicas, while acknowledging that slower conformational transitions or long‐timescale allosteric effects may require longer simulations for complete characterization.

### 5.5 Computational analyses

Post‐processing of trajectories, including translation of the protein to the center of the periodic box, was done using CPPTRAJ in AMBERTOOLS (Roe & Cheatham, 2013, 2018) and GROMACS analysis tools (Abraham et al., 2024) Root mean square fluctuations (RMSF), radius of gyration (*R*_*g*_), and hydrogen bonds, native contacts for minimum distance calculations, principal component analyses (PCA), correlation matrices, as well as long‐range contacts, were obtained using the GROMACS rmsf and gyrate module (Abraham et al., 2024), and the hbond, nativecontacts, diagmatrix, and matrix commands in the CPPTRAJ module, respectively (Roe & Cheatham, 2013, 2018). For PCA comparison between reduced and PTMed complexes, trajectories from both conditions were concatenated after structural alignment and used to construct a single covariance matrix. Eigenvectors derived from this combined trajectory were then used as a common reference subspace for projection of individual reduced and PTMed trajectories. Mode amplitude contributions were computed as the normalized sum of per‐residue *C*_α_ displacement magnitudes in each eigenvector; residues accounting for the top 50% cumulative contribution were reported. Hydrogen bonds were identified from MD trajectories using geometric criteria applied to both backbone and side‐chain atoms. A hydrogen bond was defined using a donor–acceptor distance cutoff of 3.5 Å and an angle cutoff of 30°. Hydrogen‐bond occupancy was reported as the fraction of trajectory frames of all replicates. Residue–residue dynamical correlation matrices were quantified using the normalized cross‐correlation coefficient *C*_*ij*_, computed from positional fluctuations of C_α_ atoms after removal of overall translation and rotation (Wang et al., 2011). The coefficient *C*_*ij*_ measures the extent to which motions of residues *i* and *j* are dynamically coupled, with positive values indicating correlated motion and negative values indicating anti‐correlated motion. To focus on robust and physically meaningful couplings while reducing contributions from weak correlations and statistical noise, we classified residue pairs with |*C*_*ij*_| >0.2 as strongly correlated or anti‐correlated. We also quantified PTM‐induced changes in strong residue–residue coupling by computing, for each residue, the fraction of interactions exceeding the strong‐correlation threshold (|*C*_*ij*_| >0.2). Directional changes were defined as the difference between strongly correlated and anti‐correlated fractions. These residue‐level metrics were mapped onto the reference structure for visualization. The STRIDE algorithm was used for predicting secondary structure contents (Heinig & Frishman, 2004). Prediction of catalytic pockets was performed using Caver 3.02 (Chovancova et al., 2012; Pavelka et al., 2016) gmx_MMPBSA (Valdés‐Tresanco et al., 2021) was employed to assess the interactions between OpcA and the G6PDH complex. To analyze allosteric regulations between the OpcA protein and G6PDH tetramer, we first calculated pairwise non‐bonded interactions between all residues in the three key regions of OpcA and all residues in the G6PDH tetramer. Residues that exhibited non‐bonded interaction energies >10 kcal/mol or lower than −10 kcal/mol were selected for further analysis. Subsequently, we extended this analysis to identify residues within each subunit of the G6PDH tetramer that strongly interact with gate residues (ASP33, ARG37, GLU241, and ARG243). These selected residues were used to construct flow charts that detail how long‐range interactions between OpcA and G6PDH influence the conformational states of the gates. We used Clustal Omega (Sievers et al., 2011) for multiple sequence alignment. Simple substrate docking was done via Discovery Studio Visualizer (DSV) (BIOVIA DS, 2019) All visualizations, including the rendered image of OpcA, G6PDH, and the complex, were obtained via VMD 1.9.4 (Humphrey et al., 1996) and DSV (BIOVIA DS, 2019) Protter was used to visualize the protein sequence of OpcA and G6PDH proteins (Omasits et al., 2013).

## Supporting information


**TABLE S1.** Bonded parameters of PTMed residues used in this study.
**TABLE S2.** Partial atomic charges of PTMed residues used in this study.
**FIGURE S1.** (a) Amino acid sequence of the OpcA monomer with the locations of cysteines (highlighted in yellow circles) chosen to be PTMed. Cysteine residue pairs that form disulfide bonds are highlighted by blue curved lines. (b) Amino acid sequence of the G6PDH monomer with the locations of possible residues involved in the gate configurations (highlighted in blue circles) and the active site (highlighted in red circles).
**FIGURE S2.** Structural comparisons between the predicted structures of OpcA, G6PDH monomer, G6PDH tetramer, and the OpcA‐G6PDH complex with their corresponding 3D structures available in the PDB database. The PDB ID: 9EMM contains the OpcA‐G6PDH complex from the cyanobacterial strain *Synechocystis* sp. PCC 6803, while 7SNI represents only G6PDH tetrameric complex from humans. The figure includes TM‐scores for each comparison, ranging from 0 to 1, with 0 indicating a poor match and 1 indicating a perfect match.
**FIGURE S3.** Multiple sequence alignment of (a) OpcA (OPCA_SYNE7) and (b) G6PDH protein (G6PD_SYNE7) used in this study. For (a), only OpcA from the cyanobacterial strain *Synechocystis* sp. PCC 6803 (OPCA_SYNY3), where its reaction mechanisms were previously explored, is compared. Yellow highlights represent cysteine residues of the interests and red are the not‐conversed region in the different cyanobacteria, highlighting that C398 is not conserved. Cyan and magenta in panel (b) highlights conserved residues at the upper and lower part of the gate and active site residue, respectively. A blue highlighted residue represents non‐conserved residue with a similar positively charged side chain compared to the ARG (LYS).
**FIGURE S4.** Interaction energy between the entire G6PDH subunits and reduced (gray) or PTMed (yellow) OpcA. More negative values correspond to stronger interactions.
**FIGURE S5.** Residue‐based root mean square fluctuation (RMSF) of (a–d) subunit A–D of G6PDH and (e) OpcA protein in the complex. Black and red represent the RMSF of the reduced and PTMed complex, respectively.
**FIGURE S6.** (a) Projection of MD trajectories (as a scatter plot) onto PC1 and PC2 for Subunits A–D of G6PDH tetramer in the reduced (left) and PTMed (right) systems. (b) G6PDH structure drawn by Cartoon representations with principal component displacement vectors associated with the first two principal components. (c) Representative gate conformations illustrating the physical interpretation of the sign of the PC projections and their correspondence to open and closed states.
**FIGURE S7.** Residue–residue dynamic correlation matrices (*C*_*ij*_) for the G6PDH tetramer (a: 1‐511, b: 512‐1022, c: 1023‐1533, d: 1534‐2044) and OpcA (2045‐2489). Positive (*C*_*ij*_ >0.2) and negative (*C*_*ij*_ <−0.2) values indicate correlated (red) and anti‐correlated (blue) motions, respectively. A threshold of |*C*_*ij*_| >0.2 was chosen to highlight robust dynamical couplings while minimizing noise from weak correlations.
**FIGURE S8.** Allosterically relevant interactions between OpcA and G6PDH. Key regions of OpcA (left, the gray box) are connected to G6PDH residues (dark gray flows) that exhibit strong non‐bonded interactions with other G6PDH residues (middle) involved in gate regulations (right). For interactions within G6PDH, blue, red, and orange flows represent interactions between the like‐charged residues, the oppositely charged residues, and interactions involving polar/non‐charged with charged residues, respectively. For clarity, the flow charts are grouped into three categories: (a) cases with open/closed gate conformations, (b) with closed conformations, and (c) with open or wide‐open gate conformations.

## Data Availability

The data that support the findings of this study are available from the corresponding author upon reasonable request.
