# Supplementary material for: Thiol post‐translational modifications modulate allosteric regulation of the OpcA–G6PDH complex through conformational gate control
Source: Protein Sci. 2026 Apr 13;35(5):e70561. doi: 10.1002/pro.70561 (PMC13071761; doi:10.1002/pro.70561)
Supplement: Supplementary file 1 — TABLE S1. Bonded parameters of PTMed residues used in this study. TABLE S2. Partial atomic charges of PTMed residues used in this study. FIGURE S1. (a) Amino acid sequence of the OpcA monomer with the locations of cysteines (highlighted in yellow circles) chosen to be PTMed. Cysteine residue pairs that form disulfide bonds are highlighted by blue curved lines. (b) Amino acid sequence of the G6PDH monomer with the locations of possible residues involved in the gate configurations (highlighted in blue circles) and the active site (highlighted in red circles). FIGURE S2. Structural comparisons between the predicted structures of OpcA, G6PDH monomer, G6PDH tetramer, and the OpcA‐G6PDH complex with their corresponding 3D structures available in the PDB database. The PDB ID: 9EMM contains the OpcA‐G6PDH complex from the cyanobacterial strain Synechocystis sp. PCC 6803, while 7SNI represents only G6PDH tetrameric complex from humans. The figure includes TM‐scores for each comparison, ranging from 0 to 1, with 0 indicating a poor match and 1 indicating a perfect match. FIGURE S3. Multiple sequence alignment of (a) OpcA (OPCA_SYNE7) and (b) G6PDH protein (G6PD_SYNE7) used in this study. For (a), only OpcA from the cyanobacterial strain Synechocystis sp. PCC 6803 (OPCA_SYNY3), where its reaction mechanisms were previously explored, is compared. Yellow highlights represent cysteine residues of the interests and red are the not‐conversed region in the different cyanobacteria, highlighting that C398 is not conserved. Cyan and magenta in panel (b) highlights conserved residues at the upper and lower part of the gate and active site residue, respectively. A blue highlighted residue represents non‐conserved residue with a similar positively charged side chain compared to the ARG (LYS). FIGURE S4. Interaction energy between the entire G6PDH subunits and reduced (gray) or PTMed (yellow) OpcA. More negative values correspond to stronger interactions. FIGURE S5. Residue‐based root mean [file PRO-35-e70561-s001.docx]

**SUPPORTING INFORMATION**

**Thiol post-translational modifications modulate allosteric regulation of the OpcA-G6PDH complex through conformational gate control**

Hoshin Kim^1, #, *^, Song Feng^2, #^, Pavlo Bohutskyi^2,3^, Xiaolu Li^2^ , Daniel Mejia-Rodriguez^1^, Tong Zhang^2^, Wei-Jun Qian^2^, and Margaret S. Cheung^4,5*^

^1^Physical Sciences Division, Physical and Computational Sciences Directorate, Pacific Northwest National Laboratory, Richland, Washington, USA

^2^Biological Sciences Division, Earth and Biological Sciences Directorate, Pacific Northwest National Laboratory, Richland, Washington, USA

^3^Department of Biological Systems Engineering, Washington State University, Pullman, Washington, USA

^4^Environmental Molecular Sciences Laboratory, Richland, Washington, USA

^5^University of Washington, Seattle, Washington, USA

^#^ shares co-first authorship of this article.

^*^ shares co-corresponding authorship of this article: hoshin.kim@pnnl.gov, margaret.cheung@pnnl.gov

**Table S1.** Bonded parameters of PTMed residues used in this study

| **Bond** | ***r_eq_*** | ***k_b_*** | **Torsion** | ***Φ_s_*** | ***k_Φ_*** | ***n*** |
| --- | --- | --- | --- | --- | --- | --- |
| CT - HP | 0.1090 | 284512.0 | C - N - CT - C | 0 | 1.12968 | 2 |
| N3 - CT | 0.1463 | 282001.6 |  | 0 | 1.75728 | 3 |
| S - S | 0.2038 | 138908.8 | C - CT - CT - CT | 0 | 0.65084 | 3 |
| C - N | 0.1335 | 410032.0 | C - N - CT - CT | 0 | 0.00000 | 0 |
| CT - S | 0.1810 | 189953.6 | C - CT - N - H | 180 | 4.6024 | 2 |
| C - O | 0.1229 | 476976.0 | C - N - CT - H1 | 0 | 0.00000 | 0 |
| N - CT | 0.1449 | 282001.6 | C - CT - CT - H1 | 0 | 0.65084 | 3 |
| N - H | 0.1010 | 363171.2 | C - CT - CT - S | 0 | 0.65084 | 3 |
| N3 - H | 0.1010 | 363171.2 | CT - S - S - CT | 0 | 14.6440 | 2 |
| C - O2 | 0.1250 | 548940.8 |  | 0 | 2.51040 | 3 |
| CT - CT | 0.1526 | 259408.0 | CT - C - N - CT | 180 | 10.4600 | 2 |
| CT - HC | 0.1090 | 284512.0 | CT - C - N - H | 180 | 10.4600 | 2 |
| CT - C | 0.1522 | 265265.6 | CT - CT - CT - HC | 0 | 0.65084 | 3 |
| CT - H1 | 0.1090 | 284512.0 | CT - CT - C - N | 0 | 0.83680 | 1 |
| CT - SNO | 0.1803 | 129555.1 |  | 0 | 0.83680 | 2 |
| CT - O | 0.1170 | 695768.8 |  | 0 | 1.67360 | 3 |
| SNO - NC | 0.1918 | 79743.20 | CT - CT - C - O | 0 | 0.00000 | 0 |
| OH - HO | 0.0960 | 462750.4 | CT - CT - C - O2 | 0 | 0.00000 | 0 |
| S - OH | 0.1817 | 204615.0 | CT - O2 - C - O2 | 180 | 43.9320 | 2 |
| **Angle** | ***θ_eq_*** | ***k_θ_*** | CT - CT - S - S | 0 | 1.39467 | 3 |
| C - N - CT | 121.9 | 418.40 | H - N3 - CT - C | 0 | 0.65084 | 3 |
| C - N - H | 120.0 | 418.40 | H - N - CT - CT | 0 | 0.00000 | 0 |
| CT - CT - CT | 109.5 | 334.72 | H - N3 - CT - CT | 0 | 0.65084 | 3 |
| CT - S - S | 103.7 | 569.02 | H - N - CT - H1 | 0 | 0.00000 | 0 |
| CT - C - N | 116.6 | 585.76 | H - N3 - CT - HP | 0 | 0.65084 | 3 |
| CT - CT - S | 114.7 | 418.40 | H1 - CT - CT - H1 | 0 | 0.65084 | 3 |
| CT - C - O | 120.4 | 669.44 | H1 - CT - C - N | 0 | 0.00000 | 0 |
| CT - C - O2 | 117.0 | 585.76 | H1 - CT - C - O | 0 | 3.34720 | 1 |
| CT - CT - C | 111.1 | 527.18 |  | 180 | 0.33472 | 3 |
| CT - CT - H1 | 109.5 | 418.40 | H1 - CT - C - O2 | 0 | 0.00000 | 0 |
| H - N - CT | 118.0 | 418.40 | H1 - CT - CT - S | 0 | 0.65084 | 3 |
| H - N3 - CT | 109.5 | 418.40 | H1 - CT - S - S | 0 | 1.39467 | 3 |
| H - N3 - H | 109.5 | 292.88 | HC - CT - CT - C | 0 | 0.65084 | 3 |
| H1 - CT - H1 | 109.5 | 292.88 | HC - CT - CT - HC | 0 | 0.65084 | 3 |
| H1 - CT - C | 109.5 | 418.40 | HC - CT - C - N | 0 | 0.00000 | 0 |
| H1 - CT - S | 109.5 | 418.40 | HC - CT - C - O | 0 | 3.34720 | 1 |
| HC - CT - C | 109.5 | 418.40 |  | 180 | 0.33472 | 3 |
| HC - CT - CT | 109.5 | 418.40 | HP - CT - CT - CT | 0 | 0.65084 | 3 |
| HC - CT - HC | 109.5 | 292.88 | HP - CT - CT - HC | 0 | 0.65084 | 3 |
| HP - CT - C | 109.5 | 418.40 | HP - CT - C - O2 | 0 | 0.00000 | 0 |
| HP - CT - CT | 109.5 | 418.40 | N - CT - CT - H1 | 0 | 0.65084 | 3 |
| N - CT - CT | 109.7 | 669.44 | N - CT - C - N | 180 | 1.88280 | 1 |
| N - CT - C | 110.1 | 527.18 |  | 180 | 6.61072 | 2 |
| N - CT - H1 | 109.5 | 418.40 |  | 180 | 2.30120 | 3 |
| N3 - CT - C | 111.2 | 669.44 | N - CT - C - O | 0 | 0.00000 | 0 |
| N3 - CT - CT | 111.2 | 669.44 | N - CT - C - O2 | 0 | 0.00000 | 0 |
| N3 - CT - HP | 109.5 | 418.40 | N - CT - CT - S | 0 | 0.65084 | 3 |
| O - C - N | 122.9 | 669.44 | N3 - CT - CT - CT | 0 | 0.65084 | 3 |
| O2 - C - O2 | 126.0 | 669.44 | N3 - CT - CT - HC | 0 | 0.65084 | 3 |
| CT - CT - SNO | 113.8 | 487.50 | N3 - CT - C - O2 | 0 | 0.00000 | 0 |
| CT - SNO - NC | 99.5 | 1137.0 | O - C - N - CT | 180 | 10.4600 | 2 |
| H1 - CT - SNO | 107.5 | 293.00 | O - C - N - H | 0 | 8.36800 | 1 |
| SNO - NC - O | 117.2 | 1283.1 |  | 180 | 10.4600 | 2 |
| CT - S - OH | 101.0 | 998.61 | CT - CT - SNO - NC | 180 | 0.23990 | 1 |
| S - OH - HO | 106.9 | 391.39 |  | 0 | 1.47420 | 2 |
| ***Bond***  ***r_eq_*:** Equilibrium distance (nm)  ***k_b_*:** Force Constant (kJ mol^-1^nm^-2^)  ***Angle***  **θ*_eq_*:** Equilibrium angle (deg)  ***k_θ_*:** Force Constant (kJ mol^-1^rad^-2^)  ***Torsion***  ***Φ_s_*:** Phase (deg)  ***k_Φ_*:** Force Constant (kJ mol^-1^)  ***n*:** Multiplicity (unitless) | | | H1 - CT - SNO - NC | 180 | 1.46130 | 3 |
|  |  |  | CT - SNO - NC - O | 0 | 1.07690 | 2 |
|  |  |  |  | 180 | 27.2930 | 2 |
|  |  |  |  | 180 | 2.72140 | 3 |
|  |  |  |  | 180 | 0.50325 | 4 |
|  |  |  | C - CT - CT - SNO | 0 | 0.65084 | 3 |
|  |  |  | H1 - CT - CT - SNO | 0 | 0.65084 | 3 |
|  |  |  | N - CT - CT - SNO | 0 | 0.65084 | 3 |
|  |  |  | CT - S - OH - HO | 0 | 2.34510 | 1 |
|  |  |  |  | 0 | 9.36040 | 2 |
|  |  |  | CT - CT - S - OH | 180 | 8.02970 | 1 |
|  |  |  |  | 0 | 0.52192 | 2 |
|  |  |  |  | 0 | 4.95200 | 3 |
|  |  |  | H1 - CT - S - OH | 0 | 1.39467 | 3 |

**Table S2.** Partial atomic charges of PTMed residues used in this study

| **Glutathionylated Cysteine (CGL)** | | | **Nitrosylated Cysteine (SNC)** | | |
| --- | --- | --- | --- | --- | --- |
| **Atom Name** | **Atom Type** | **Partial Charge** | **Atom Name** | **Atom Type** | **Partial Charge** |
| N | N | -0.4157 | N | N | -0.4157 |
| H | H | 0.2719 | H | H | 0.2719 |
| CA | CT | 0.0429 | CA | CT | -0.0218 |
| HA | H1 | 0.0766 | HA | H1 | 0.0758 |
| CB | CT | -0.079 | CB | CT | 0.4533 |
| HB1 | H1 | 0.0910 | HB1 | H1 | -0.0678 |
| HB2 | H1 | 0.0910 | HB2 | H1 | -0.0678 |
| SG | S | -0.1081 | SG | SNO | -0.1960 |
| N2 | N3 | -0.8689 | ND | NC | 0.0855 |
| H21 | H | 0.3395 | OE | O | -0.1468 |
| H22 | H | 0.3395 | C | C | 0.5972 |
| H23 | H | 0.3395 | O | O | -0.5679 |
| CA2 | CT | 0.2825 | **Sulfenylated Cysteine (CSO)** | | |
| HA2 | HP | 0.0238 | **Atom Name** | **Atom Type** | **Partial Charge** |
| CB2 | CT | 0.0122 | N | N | -0.4157 |
| HB21 | HC | 0.0095 | H | H | 0.2719 |
| HB22 | HC | 0.0095 | CA | CT | 0.0063 |
| CG2 | CT | -0.0498 | HA | H1 | 0.0907 |
| HG21 | HC | 0.0086 | CB | CT | 0.1531 |
| HG22 | HC | 0.0086 | HB1 | H1 | 0.0141 |
| C2 | C | 0.6306 | HB2 | H1 | 0.0141 |
| OC21 | O2 | -0.5382 | SG | S | -0.0683 |
| OC22 | O2 | -0.5382 | OD | OH | -0.4757 |
| CD2 | C | 0.5449 | HD | HO | 0.3801 |
| OE2 | O | -0.5536 | C | C | 0.5972 |
| N3 | N | -0.4157 | O | O | -0.5679 |
| H3 | H | 0.2719 |  | | |
| CA3 | CT | 0.0429 |  |  |  |
| HA3 | H1 | 0.0766 |  |  |  |
| CB3 | CT | -0.079 |  |  |  |
| HB31 | H1 | 0.0910 |  |  |  |
| HB32 | H1 | 0.0910 |  |  |  |
| SG3 | S | -0.1081 |  |  |  |
| C3 | C | 0.5973 |  |  |  |
| O3 | O | -0.5679 |  |  |  |
| N4 | N | -0.3821 |  |  |  |
| H4 | H | 0.2681 |  |  |  |
| CA4 | CT | -0.2493 |  |  |  |
| HA41 | H1 | 0.1056 |  |  |  |
| HA42 | H1 | 0.1056 |  |  |  |
| C4 | C | 0.7231 |  |  |  |
| OC41 | O2 | -0.7855 |  |  |  |
| OC42 | O2 | -0.7855 |  |  |  |
| C | C | 0.5973 |  |  |  |
| O | O | -0.5679 |  |  |  |


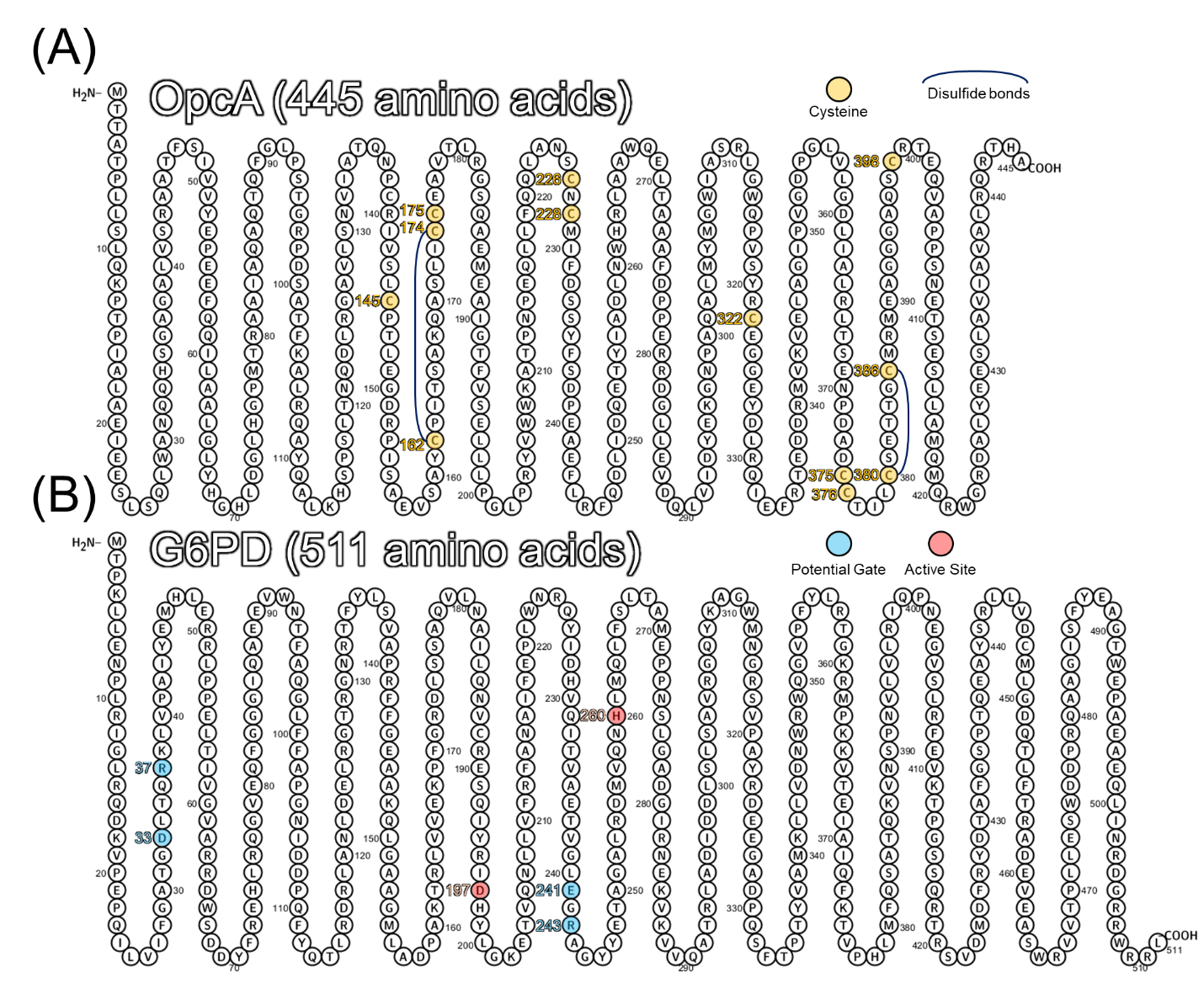


**Figure S1.** (A) Amino acid sequence of the OpcA monomer with the locations of cysteines (highlighted in yellow circles) chosen to be PTMed. Cysteine residue pairs that form disulfide bonds are highlighted by blue curved lines. (B) Amino acid sequence of the G6PDH monomer with the locations of possible residues involved in the gate configurations (highlighted in blue circles) and the active site (highlighted in red circles).


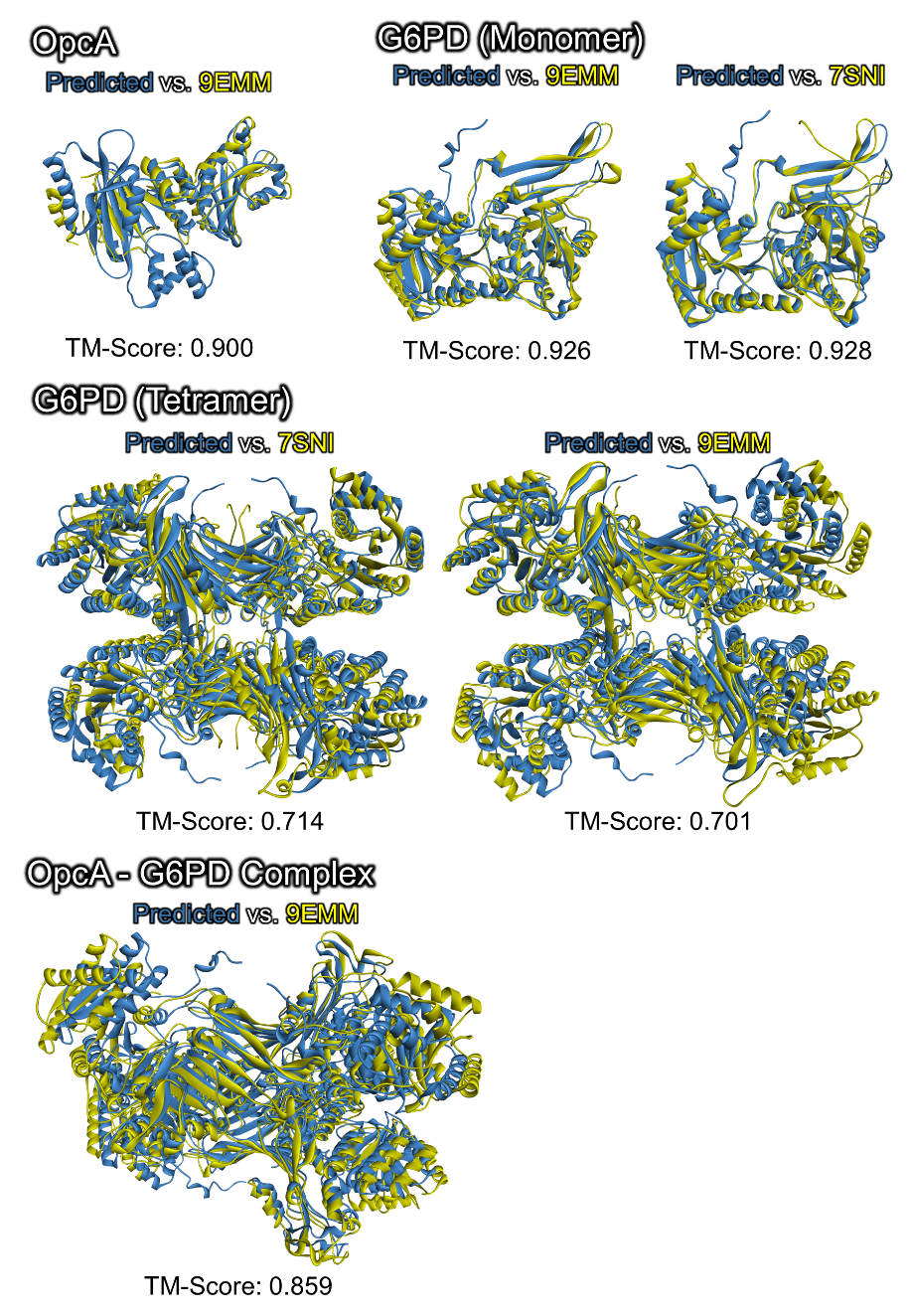


**Figure S2.** Structural comparisons between the predicted structures of OpcA, G6PDH monomer, G6PDH tetramer, and the OpcA-G6PDH complex with their corresponding 3D structures available in the PDB database. The PDB ID: 9EMM contains the OpcA-G6PDH complex from the cyanobacterial strain *Synechocystis* sp. PCC 6803, while 7SNI represents only G6PDH tetrameric complex from humans. The figure includes TM-scores for each comparison, ranging from 0 to 1, with 0 indicating a poor match and 1 indicating a perfect match.


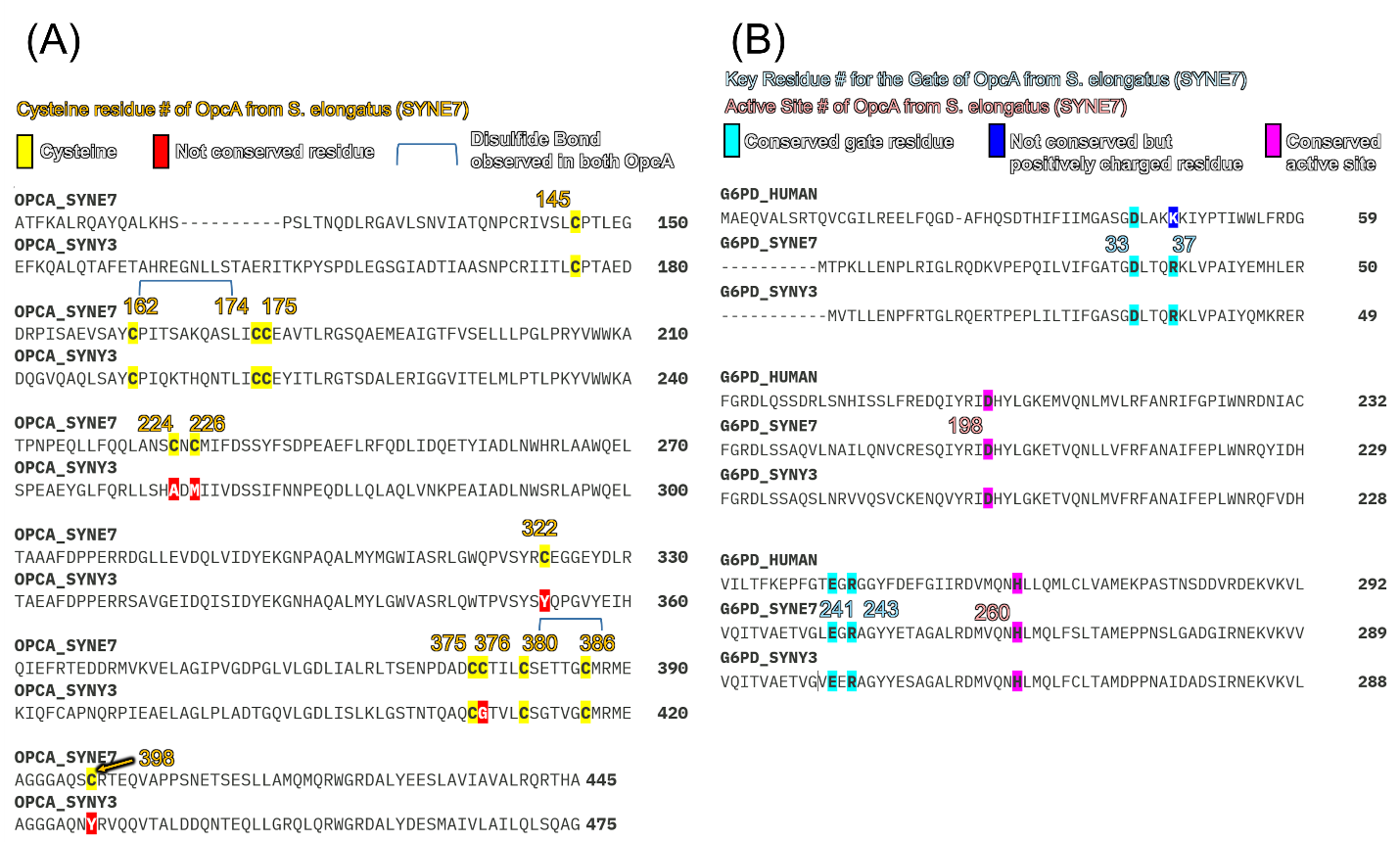


**Figure S3**. Multiple sequence alignment of (A) OpcA (OPCA_SYNE7) and (B) G6PDH protein (G6PD_SYNE7) used in this study. For (A), only OpcA from the cyanobacterial strain *Synechocystis* sp. PCC 6803 (OPCA_SYNY3), where its reaction mechanisms were previously explored, is compared. Yellow highlights represent cysteine residues of the interests and red are the not-conversed region in the different cyanobacteria, highlighting that C398 is not conserved. Cyan and magenta in panel (B) highlights conserved residues at the upper and lower part of the gate and active site residue, respectively. A blue highlighted residue represents non-conserved residue with a similar positively charged side chain compared to the ARG (LYS).


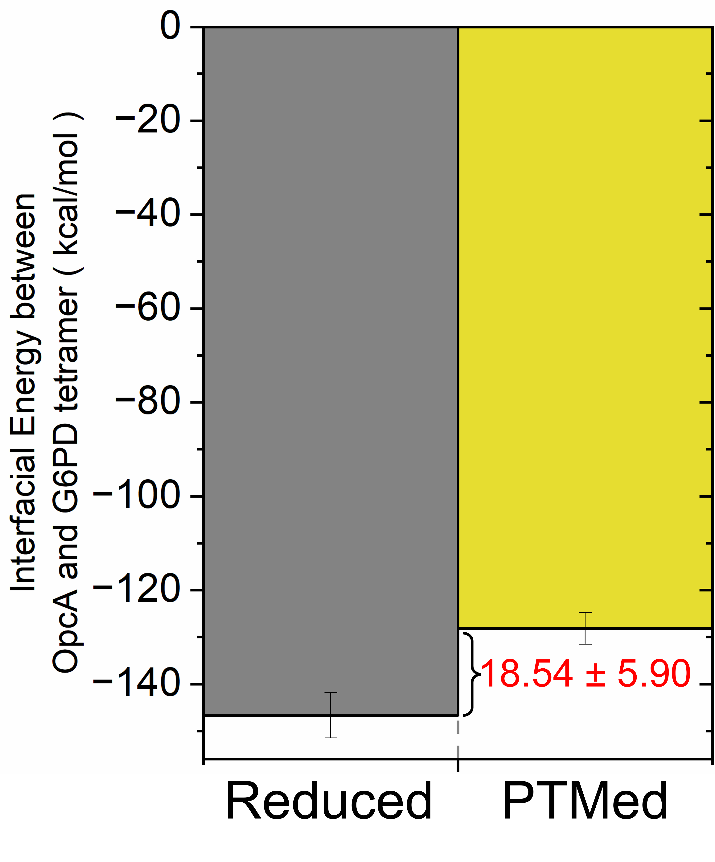


**Figure S4.** Interaction energy between the entire G6PDH subunits and reduced (gray) or PTMed (yellow) OpcA. More negative values correspond to stronger interactions.


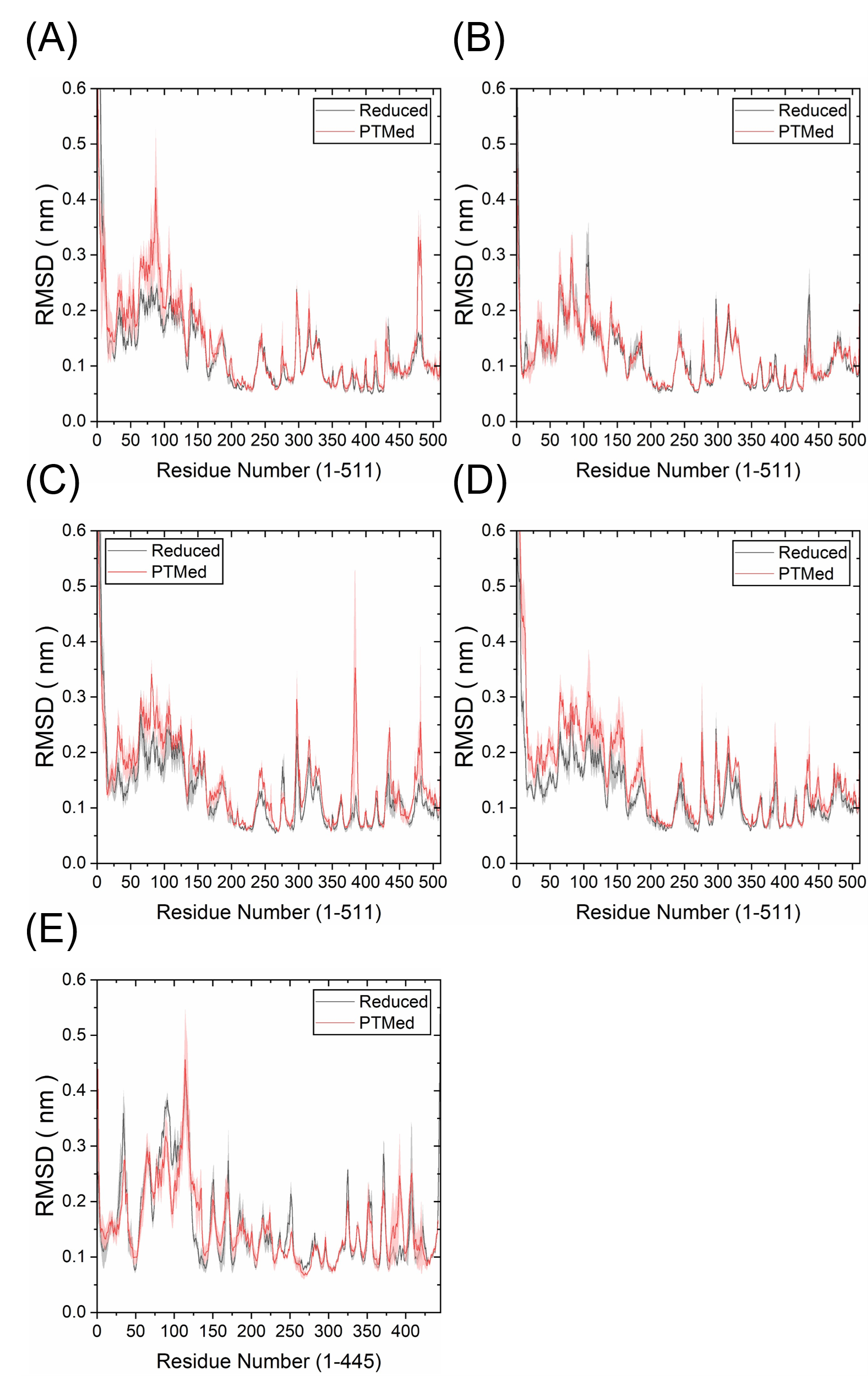


**Figure S5.** Residue-based root mean square fluctuation (RMSF) of (A-D) subunit A-D of G6PDH and (E) OpcA protein in the complex. Black and red represent the RMSF of the reduced and PTMed complex, respectively.


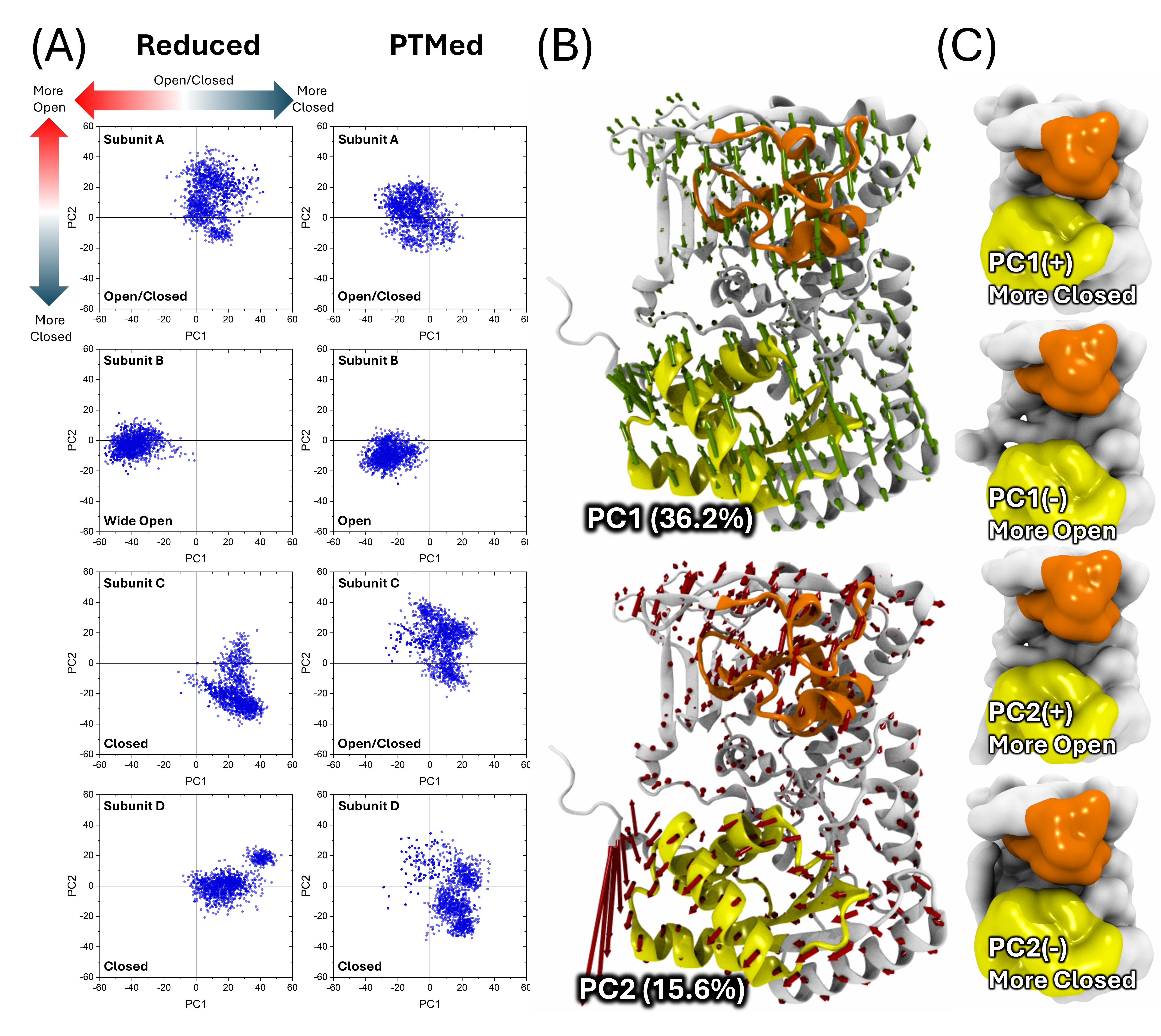


**Figure S6.** **(A)** Projection of MD trajectories (as a scatter plot) onto PC1 and PC2 for Subunits A–D of G6PDH tetramer in the reduced (left) and PTMed (right) systems. **(B)** G6PDH structure drawn by Cartoon representations with principal component displacement vectors associated with the first two principal components. **(C)** Representative gate conformations illustrating the physical interpretation of the sign of the PC projections and their correspondence to open and closed states.


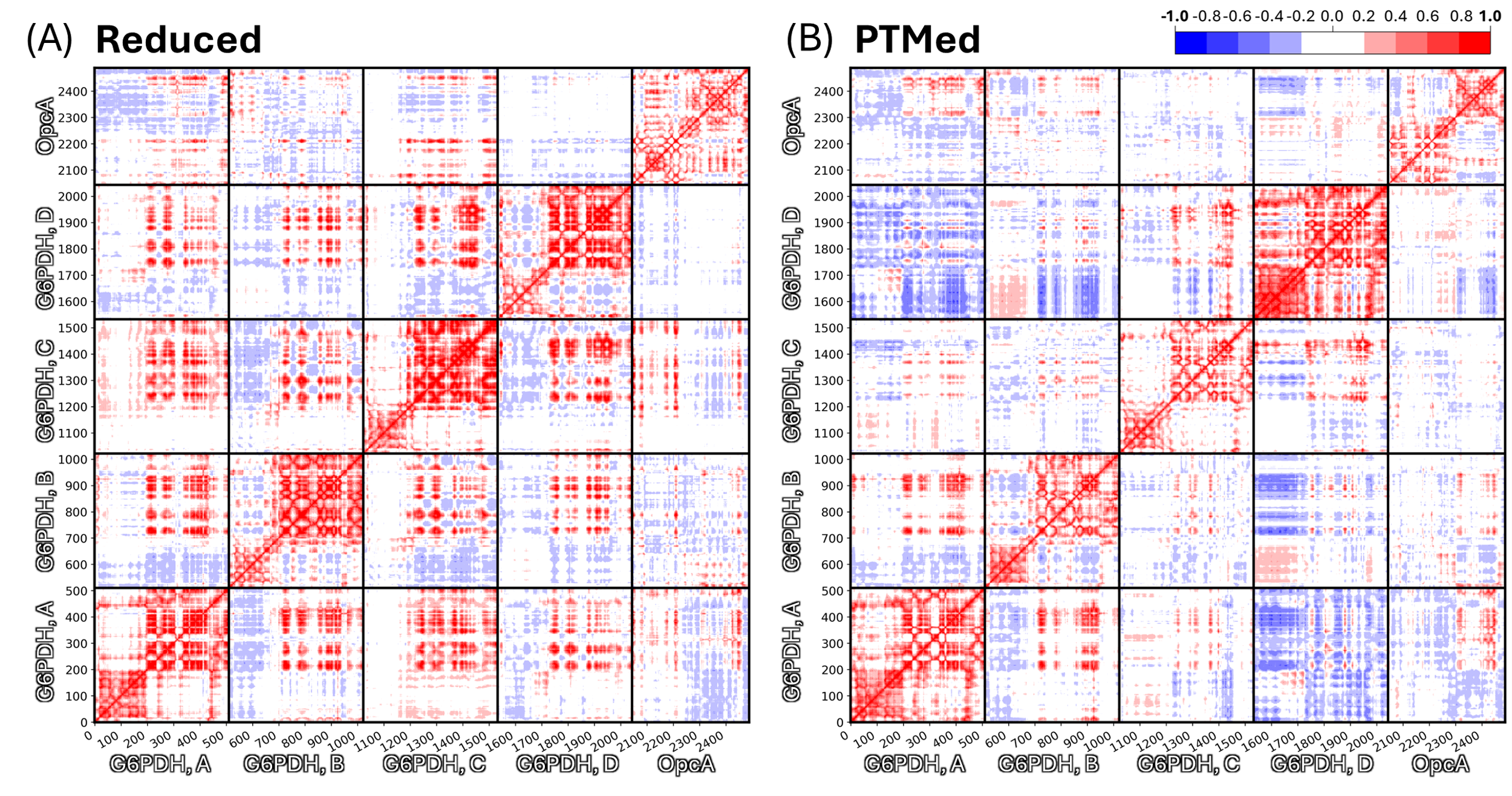


**Figure S7.** Residue–residue dynamic correlation matrices (*C_ij_*) for the G6PDH tetramer (A: 1-511, B: 512-1022, C: 1023-1533, D: 1534-2044) and OpcA (2045-2489). Positive (*C_ij_* > 0.2) and negative (*C_ij_* < -0.2) values indicate correlated (red) and anti-correlated (blue) motions, respectively. A threshold of |*C_ij_*| > 0.2 was chosen to highlight robust dynamical couplings while minimizing noise from weak correlations.


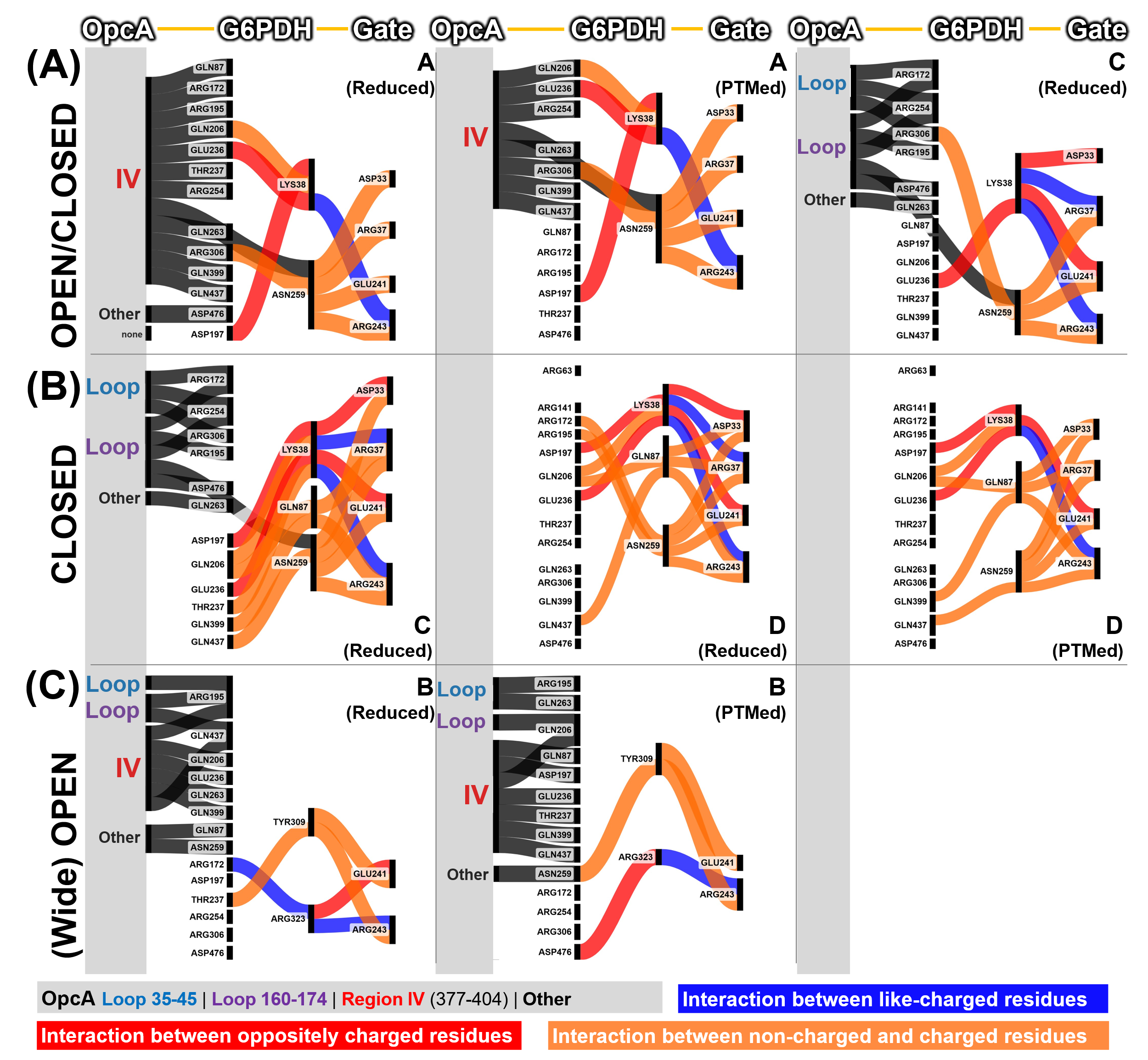


**Figure S8.**  Allosterically relevant interactions between OpcA and G6PDH. Key regions of OpcA (left, the gray box) are connected to G6PDH residues (dark gray flows) that exhibit strong non-bonded interactions with other G6PDH residues (middle) involved in gate regulations (right). For interactions within G6PDH, blue, red, and orange flows represent interactions between the like-charged residues, the oppositely charged residues, and interactions involving polar/non-charged with charged residues, respectively. For clarity, the flow charts are grouped into three categories: (A) cases with open/closed gate conformations, (B) with closed conformations, and (C) with open or wide-open gate conformations.
